# Genome-wide SNPs and candidate genes underlying the genetic variations for protein and amino acids in pearl millet (*Pennisetum glaucum*) germplasm

**DOI:** 10.1007/s00425-024-04495-y

**Published:** 2024-07-27

**Authors:** Satbeer Singh, Chandra Bhan Yadav, Nelson Lubanga, Matthew Hegarty, Rattan S. Yadav

**Affiliations:** 1 Institute of Biological Environmental and Rural Sciences (IBERS), Aberystwyth University, Aberystwyth, SY23 3EE UK; 2 Division of Agrotechnology, Council of Scientific and Industrial Research (CSIR) - Institute of Himalayan Bioresource Technology, Palampur, Himachal Pradesh 176 061 India; 3 Department of Genetics, Genomics, and Breeding, NIAB-EMR, East Malling, ME19 6BJ UK

**Keywords:** Essential amino acids, GWAS, Malnutrition, Pearl millet, PMiGAP, Total protein

## Abstract

**Main conclusion:**

A total of 544 significant marker-trait associations and 286 candidate genes associated with total protein and 18 amino acids were identified. Thirty-three candidate genes were found near the strong marker trait associations (– log_10_*P* ≥ 5.5).

**Abstract:**

Pearl millet (*Pennisetum glaucum*) is largely grown as a subsistence crop in South Asia and sub-Saharan Africa. It serves as a major source of daily protein intake in these regions. Despite its importance, no systematic effort has been made to study the genetic variations of protein and amino acid content in pearl millet germplasm. The present study was undertaken to dissect the global genetic variations of total protein and 18 essential and non-essential amino acids in pearl millet, using a set of 435 K Single Nucleotide Polymorphisms (SNPs) and 161 genotypes of the Pearl Millet Inbred Germplasm Association Panel (PMiGAP). A total of 544 significant marker-trait associations (at *P* < 0.0001; – log_10_*P* ≥ 4) were detected and 23 strong marker-trait associations were identified using Bonferroni’s correction method. Forty-eight pleiotropic loci were found in the genome for the studied traits. In total, 286 candidate genes associated with total protein and 18 amino acids were identified. Thirty-three candidate genes were found near strongly associated SNPs. The associated markers and the candidate genes provide an insight into the genetic architecture of the traits studied and are going to be useful in breeding improved pearl millet varieties in the future. Availabilities of improved pearl millet varieties possessing higher protein and amino acid compositions will help combat the rising malnutrition problem via diet.

**Supplementary Information:**

The online version contains supplementary material available at 10.1007/s00425-024-04495-y.

## Introduction

According to an estimate by the FAO, around 2.5 billion people globally suffer from nutritional deficiencies and show symptoms of malnutrition (https://www.who.int/news-room/fact-sheets/detail/malnutrition). Of these, protein malnutrition alone impacts around one billion vegetarian and vegan populations in Sub-Saharan Africa and South Asia (Wu et al. 2014). Food and Agriculture Organization celebrated the year 2023 as an “International Year of Millets" across the world to raise awareness of millets as a future crop to foster food and nutritional security. Pearl millet (*Pennisetum glaucum*) is crucial in enhancing nutritional security because its grain naturally possesses a range of nutritional and health-benefitting traits (Hassan et al. 2021). In addition, pearl millet is a climate-resilient crop that grows well in arid and semi-arid regions with low soil fertility, high temperatures and low rainfall where other crops tend to fail. Its ability to thrive in such conditions contributes to food and nutritional security in the face of climate change. Further, characteristics like high complex carbohydrate levels, high fibre, greater α-amylase activity, low glycaemic index and gluten-free possessed by pearl millet make it an ideal model crop for use in functional food industries (Saleh et al. 2013; Yadav et al. 2022).

**Fig. 1:**
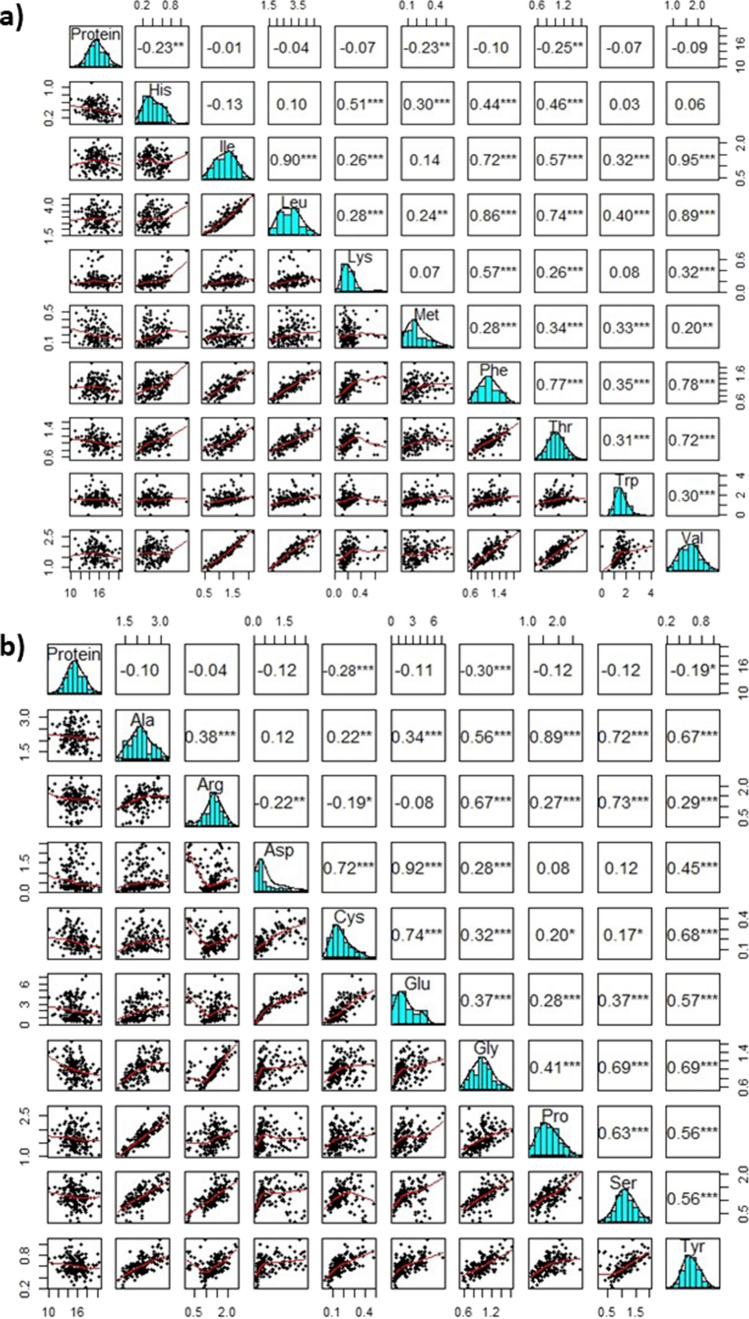
Distribution (diagonal) and association among protein and essential (**a**) as well as non-essential amino acids (**b**). The below diagonal are the scatter plots of 161 genotypes with the best fit red line. Above diagonals are the correlation coefficients superscripts with significance levels *P ≤ 0.05, **P ≤ 0.01 and ***P ≤ 0.001

Protein content and amino acid compositions are essential ingredients in determining the nutritional qualities of any food grain (Shewry 2007). The total protein content in pearl millet varieties has been reported to range from 10 to 13% and is higher than major cereals like maize, rice, wheat and sorghum (Ejeta et al. 1987; Anitha et al. 2020; Tomicic et al. 2022). Recently, the pearl millet germplasm (PMiGAP panel) was studied and has been found to possess even higher concentrations of protein (10–20%) (data not shown) than reported previously using cultivated varieties. This indicated the PMiGAP resource has higher potentials to improve the total protein of commercial pearl millet varieties. In addition to total protein, the quality of protein is equally important and is determined by the presence of essential amino acids such as lysine, leucine, methionine, tryptophan, and threonine (Fig. 1a) that the human body cannot synthesize and need to be obtained through food. In plants, the amino acids essentially serve as precursors for hormones, important secondary metabolites, osmolytes, stress response, alternative energy sources, seed germination and development (Zhao et al. 2009).

The genomic regions and putative genes controlling metabolic pathways for amino acids have been thoroughly investigated in crops like *Arabidopsis* (Angelovici et al. 2013), rice (Huang et al. 2019), sorghum (Kimani et al. 2020) and maize (Deng et al. 2017). Furthermore, many of the well-characterized genes like *floury-2*, *high-lysine*, and *opaque-2* have been utilized in breeding programs to develop high-lysine content varieties in maize (Deng et al. 2017). To the best of our knowledge, no such studies have been reported in pearl millet. Systematic studies to capture natural variation, underlying genomic regions, putative genes, and to incorporate these in breeding programs are therefore the needs of the hour in pearl millet.

Genome-wide association study (GWAS) is an effective method of exploring the genetic basis of complex nutritional traits using naturally occurring genetic diversity (Korte and Farlow 2013; Cortes et al. 2021). GWAS provides high resolution by evaluating many alleles at a single locus accumulated as a result of recombinational events over years in natural populations. It overcomes the limitation of linkage mapping, which captures a small number of loci from biparental populations (Rafalski 2010). In previous GWAS studies, we successfully reported the identification of genomic regions for anti-oxidant activities and starch traits in pearl millet (Yadav et al. 2021, 2022) using 78 K genome-wide SNPs derived from genome re-sequencing of the PMiGAP entries (Varshney et al. 2017). In the present study, a larger set of SNPs (435 K) were identified based on a recently published improved pangenome sequence (Ramu et al. 2023) which provided both an increased genomic coverage as well as reduced confounding errors compared to the reference genome. The aims of this study was to identify genome-wide regions and candidate genes for 19 different traits of protein and amino acid compositions.

## Materials and methods

### Plant material

A total of 161 genotypes were selected randomly from within a set of 340 genotypes in the pearl millet inbred germplasm association panel PMiGAP (Sehgal et al. 2015) for use in the present study. This set of 161 genotypes comprised landraces (*n* = 131), improved cultivars (*n* = 10), and breeding lines (*n* = 20) from 23 different countries (Online Resource S1). The seeds of the individual entries of the PMiGAP were multiplied as previously described (Upadhyaya et al. 2008; Ramya et al. 2018; Yadav et al. 2022) and were obtained from the International Crops Research Institute for the Semi-Arid Tropics (ICRISAT, Patancheru, India) for use in this study.

### Protein and amino acid analysis

The grain samples were analysed for total nitrogen (N) by a rapid combustion method using a LECO FP-528 analyser (LECO Corp., St. Joseph, MI, USA) by following the manufacturer's recommendations. Nitrogen combustion was performed at 850 °C temperatures in the tubes and 750 °C in the reduction heater. Two analytical-grade reference materials (minimum assay 99.9%), L-lysine-HCl (15.32% *N*) and L-tryptophan (13.71% *N*), as well as soy flour (8.55% *N*) were used to monitor nitrogen recovery. Ethylene diamine tetra-acetic acid (EDTA) (9.56 ± 0.04% *N*) was used for nitrogen calibration for the Dumas method. The protein content was determined using the nitrogen-to-protein factor *N* × 6.25 and reported as a percent of the grain weight.

A chloroform–methanol mixture (2:1, v:v) was used to extract and remove lipids. The remaining samples after the lipid extraction were air-dried and subsequently hydrolyzed (Boulos et al. 2020). About 50 mg samples were hydrolyzed with hydrochloric acid at 110 °C for 24 h followed by cooling, neutralized with sodium hydroxide and dried in a speed-vacuum concentrator. Ethanol:water:triethylamine:phenyl-isothiocyanate (7:1:1:1, by vol.) was used to derivatize the samples followed by drying and subsequent re-suspension of pellets in a sodium phosphate buffer containing 5% acetonitrile and filtered before transferred into LC vials. To enable precise identification and quantification, a series of dilutions of Amino Acid Standard H standard (Thermo Scientific) was run alongside the samples to allow accurate identifications and quantification. Samples were run on a C18 analytical Pico-Tag amino acid analysis column (3.9 × 150 mm) at 38 °C on a Vanquish HPLC equipped with a diode array detector set at 254 nm. Separation was achieved by a gradient of eluent A (150 mM sodium acetate, 0.05% NEt3 and 6% acetonitrile (ACN), adjusted to pH 6.4 with 10% acetic acid) and eluent B (acetonitrile:water, 6:4, v/v) starting at 0%B, increasing to 20% B within 5.5 min, linearly to 46% B at 10 min, changing to 100% B until 10.5 min, 2 min isocratic, and back to 0% B within 0.5 min following re-equilibration with eluent A for 20 min.

For tryptophan analysis, after neutralizing with hydrochloric acid samples were centrifuged to remove particulates and the supernatant was diluted 1/10 in water before being transferred to LC vials. For precise identification and quantification, a tryptophan dilution series was conducted alongside the samples, on a Vanquish HPLC with a fluorescence detector, samples were run on the same C18 column at 30 °C. The isocratic sodium acetate buffer containing 10% acetonitrile was the mobile phase that was employed. During the 10-min run duration, a 280 nm excitation wavelength and a 340 nm emission wavelength were employed. The *r*^2^ value for the calibration curves for all measured amino acids at concentrations between 1.82 and 750 µM (0.43 and 16.2 µM for Trp) are given in supplementary material (Online Resource S2).

### Genotyping and Single Nucleotide Polymorphism (SNPs)

To perform genetic structure and GWAS analysis, re-sequencing data of 161 genotypes were derived from the publicly available dataset (Ramu et al. 2023). A total of 30 million SNPs were detected using this recently published improved pangenome reference pipeline (Ramu et al. 2023). Further, a final set of 435 K SNPs of 161 genotypes was filtered to identify unlinked SNPs (*r*^2^ < 0.8) in a window of 50 bp, minor allele frequency of more than 0.05, and missing rate of less than 10% in PLINK (Purcell et al. 2007).

### Population structure

The population structure for ancestry co-inheritance in unrelated individuals of the PMiGAP was assessed using the finally filtered set of 435 K SNPs through the ADMIXTURE algorithm at the PLINK platform (Alexander and Lange 2011). The cross-validation errors were computed for *K* values ranging from *K* = 2 to *K* = 10 to determine the actual number of ancestral admixtures. Additionally, principal component analysis and phylogenetic clustering were carried out to assess the genome-wide variation and genetic relationship among different sub-populations within the PMiGAP. A total of ten principal components were observed (Online Resource S3) and plotting was done on the first three principal components explaining the highest cumulative variance.

### GWAS analysis

The genome-wide association analysis was carried out for protein content and all amino acid parameters using multi-locus Fixed and random model Circulating Probability Unification (FarmCPU) model in Genome Association and Prediction Integrated Tool (GAPIT) function on Rstudio (Wang and Zhang 2021; Posit Team 2023). The Manhattan plots and combined -log_10_(*P* values) plots were created using CMplots and ggplots functions in RStudio. A threshold for the significance of SNP markers was set at *P* value < 0.0001 (– log_10_*P* ≥ 4.0). However, to reduce the false positive at maximum capacity for the traits where a large number of positives were observed even at – log_10_*P* > 4.0, the cutoff level for significant markers was established using a modified multiple-test Bonferroni's correction (Kimani et al. 2020).

### In silico candidate gene analysis

The SnpEff eff (Version 4.3.1t) package (Cingolani et al. 2012) was used to detect candidate genes on the *Pennisetum glaucum* reference genome (Tift23D2B1-P1-P5) within regions around 50 kb around significant SNPs to avoid the expected linkage disequilibrium (LD) decay in this highly cross-pollinated crop. Using the SnpEff eff package, a custom database was constructed using the GFF file of the Tift reference genome (Tift23D2B1-P1-P5). The upstream and downstream regions surrounding each strongly linked SNP were investigated to identify and propose potential candidate genes. In addition, each region of interest with potential candidate genes was subjected to a Basic Local Alignment Search Tool (BLAST) search of the NCBI database against the National Center for Biotechnology Information (NCBI) nr database.

## Results

### Phenotypic variation

The PMiGAP GWAS panel showed the presence of significant natural genetic variation for all characteristics studied, including protein quantity, and quality represented by essential and non-essential amino acids (Fig. 1). The detailed patterns of diversity of protein and amino acids in PMiGAP are given in our previous report (Singh et al. 2024). In brief, protein content determined as a percentage of the grain weight ranged from 10.1% to 20.3% with an average of 15.5%, and amino acids were observed as grams per 100 g of protein and varied from 0.03 g to 7.20 g in PMiGAP. Among the amino acids, glutamic acid (0.21–7.20 g) and leucine (1.59–4.81 g) were the most abundant in the PMiGAP followed by tryptophan (0.01–4.00 g), alanine (1.20–3.26 g), proline (1.06–2.79 g) and valine (0.84–2.74 g). A normal frequency distribution was observed for almost all the traits studied and presented on the diagonal of Fig. 1a (essential amino acids) and Fig. 1b (non-essential amino acids). The above diagonal values given in Fig. 1 showed a highly significant correlation among almost all the amino acids except pairs of His vs Ile/leu/Trp/Val, Ile vs Met, Lys vs Met/Trp, Asp vs Ala/Pro/Ser/Arg and Arg vs Cys/Glu. Notably, the protein content was observed negatively correlated with all the amino acids.

### Marker distribution and population structure

**Fig. 2:**
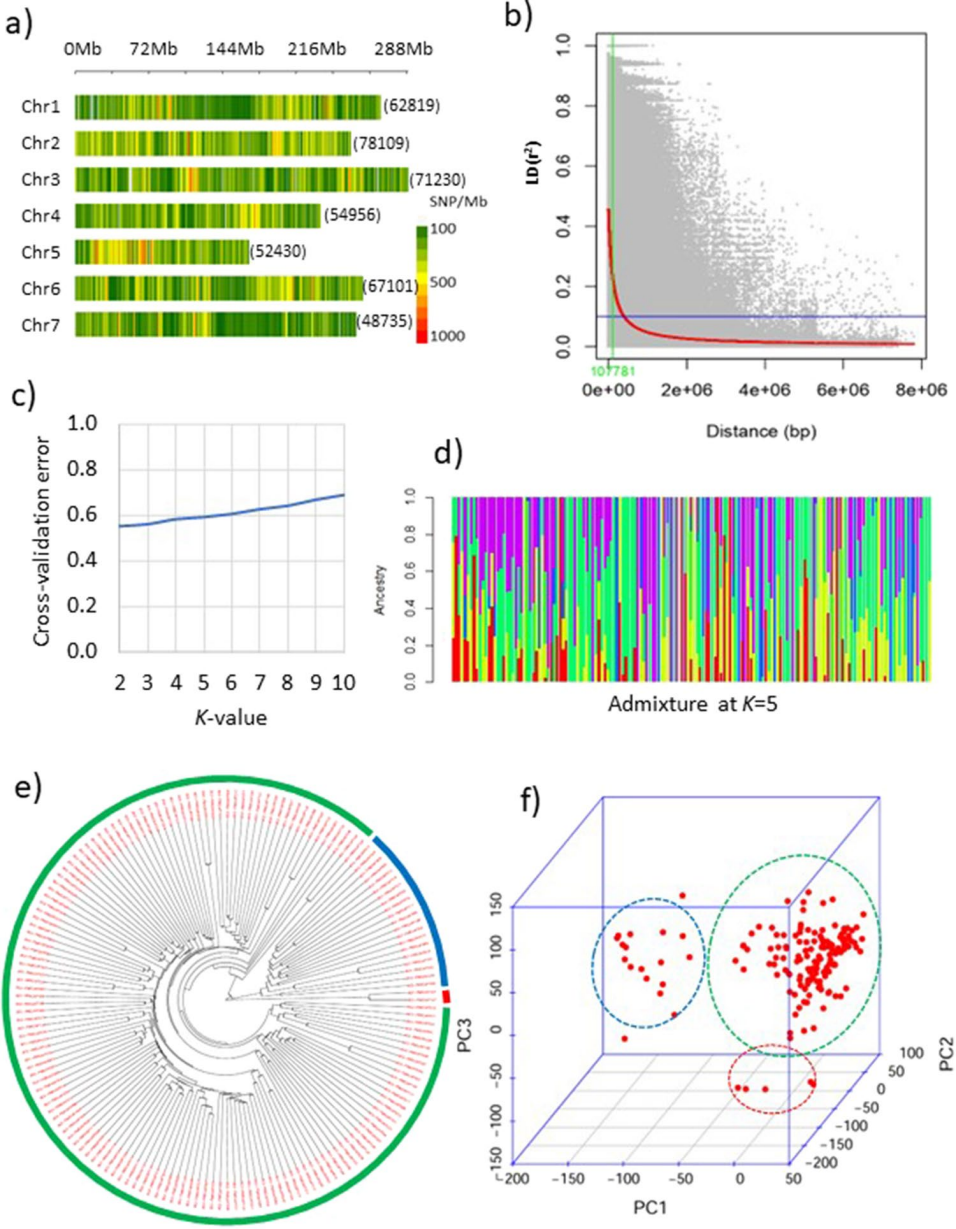
Population structure of 161 genotypes based on 435 K SNPs. **a** Marker distribution. **b** LD decay (red line) and the distance (green) where 50% LD decay met. **c** Cross-validation error over *K*-2 to *K*-10. **d** Ancestral admixture at random *K*-10. **e** Neighbor-joining tree. **f** Plot of first three PCs

A set of filtered 435 K SNPs (*r*^2^ ≤ 0.8, minor allele frequency (MAF) > 0.05, and < 10% missing rate) was used for such analysis. These SNPs randomly covered a total of 1859 Mb genome size with an average of 266 Mb per chromosome ranging from 170 Mb (Chr5) to 325 Mb (Chr3) (Fig. 2a). The highest number of SNPs were present on chromosome 2 and the least was on chromosome 7 (Online Resource S4). The chromosome density was highest in chromosome 5 (309 SNPs/Mb) and least for chromosome 7 (177 SNPs/Mb). The estimations of LD in the studied 161 genotypes of PMiGAP for the genome-wide SNPs rapidly decreased up to *r*^2^ = 0.1 with an increase in distance under 500 Kb (Fig. 2b, Online Resource S5). The 50% decay of the maximum LD met at 107781 bp ~ 100 Kb (Fig. 2b). The total genome size (1.79 Gb) was divided by the distance (107,781 bp) of 50% LD decay to estimate the number of independent tests (16,607). Finally, Bonferroni’s correction threshold of – log_10_*P* ≥ 5.5 was computed for type I error by assuming 0.05 probability over 16,607 independent tests. Further, the population structure was examined with the ancestral admixture algorithm. The negligible changes in the cross-validation error over different *k* values ranging from 2 to 10 (Fig. 2c and d) suggested that PMiGAP is an unstructured population at a molecular level. This was again confirmed with the neighbour-joining tree-based clustering (Fig. 2e) and principal component analysis (Fig. 2f), which revealed that most of the genotypes (139 out of 161) remained ungrouped and formed a large cluster. The results pertaining to marker distribution and population structure indicated that the set of filtered SNPs and sub-set of 161 genotypes from the PMiGAP panel were suitable for further marker-trait association analysis.

### Significant marker-trait associations

**Fig. 3:**
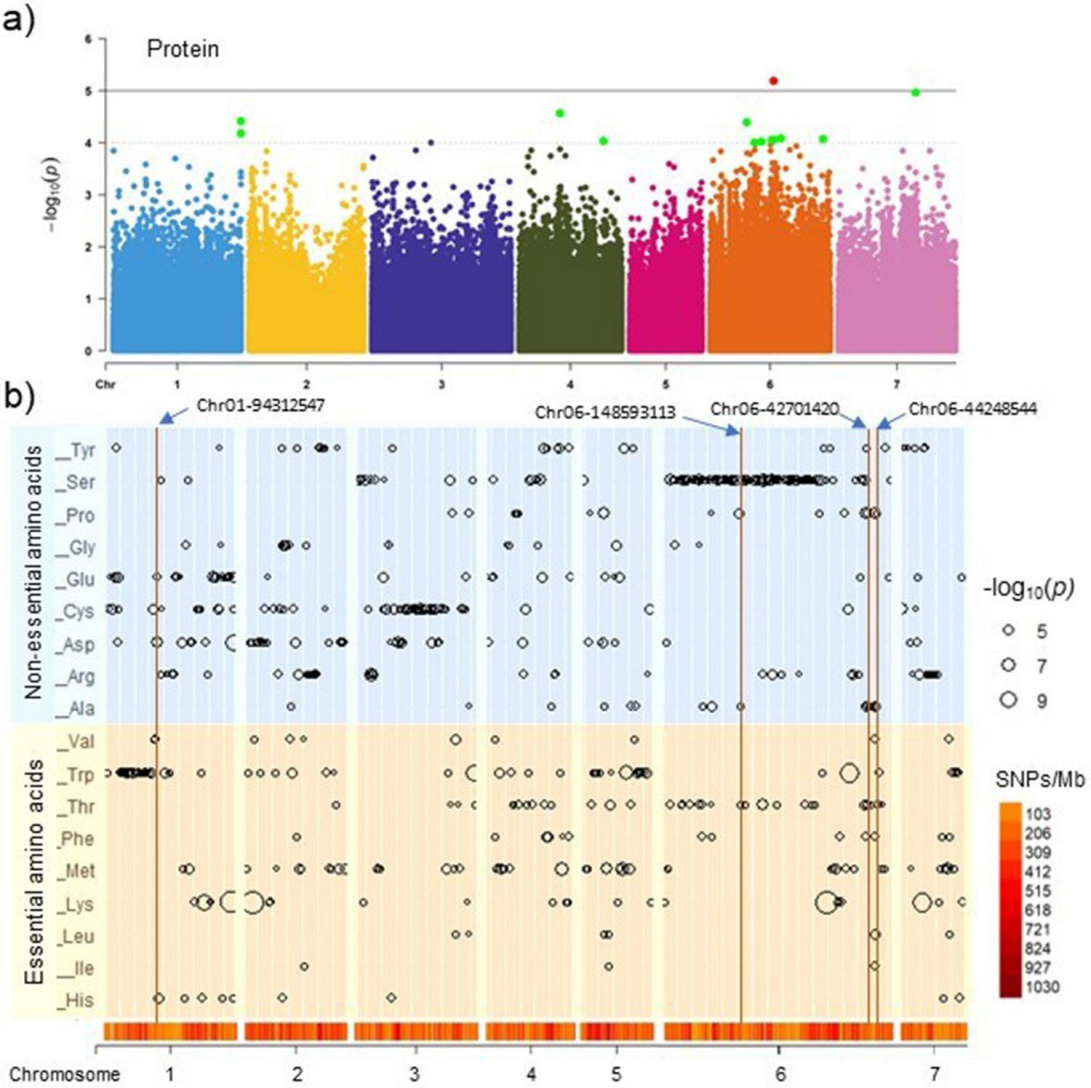
Manhattan plot for protein (**a**) and distribution plot of significant marker-trait associations (– log_10_*P* > 4 or *P* value < 0.0001) identified for eighteen essential and non-essential amino acids traits (**b**). The markers’ positions in plot ‘**b**’ are represented by circles and the size of the circles is proportionate to the *P* values. The standing bars **b** represent pleiotropic loci for more than three traits. The heat density map on the x-axis represents the distribution of the total markers

To explain the genetic basis of variation underlying the protein and amino acid traits in pearl millet, a genome-wide association study was carried out using the Fixed and random model Circulating Probability Unification model. A total of 544 SNPs (range from 3 SNPs for isoleucine to 106 SNPs for serine) were associated with all traits at a *P* value < 0.0001 (– log_10_*P* ≥ 4) (Fig. 3, Online Resource S6 and S7). The highest number of marker-trait associations (169 SNPs) were observed at chromosome 6, while the least at chromosome 7 (41 SNPs).

To reduce false positives and to increase confidence in marker-trait associations, a multi-testing Bonferroni’s correction threshold (– log_10_*P* ≥ 5.5) was applied, and finally a total of 23 strong marker-trait associations were observed for nine traits viz., lysine (5 SNPs: Chr02-121838429, Chr06-172222184, Chr01-94231924, Chr07-196098235 and Chr01-90166787), serine (5 SNPs: Chr06-154131992, Chr06-147646969, Chr04-24900886, Chr06-154333801 and Chr06-141278568), tryptophan (3 SNPs: Chr06-249278794, Chr03-72850957 and Chr05-56191227), aspartic acid (3 SNPs: Chr01-94312547, Chr01-217678759 and Chr03-237566752), methionine (2 SNPs: Chr05-28823300 and Chr04-6908911), proline (2 SNPs: Chr06-42701420 and Chr05-140112743), arginine (1 SNP: Chr03-152794146), cystine (1 SNP: Chr03-239439074) and glutamic acid (1 SNP: Chr01-91831977). These strong marker-trait associations were found highest at chromosome-6 (7 SNPs) followed by chromosome-1 (6 SNPs), chromosome-3 (4 SNPs) and chromosome-5 (3 SNPs).

A total of 48 loci were pleiotropic for at least two to a maximum of six traits. The highest 19 pleiotropic loci were present on chromosome-6 followed by six loci each on chromosome-1, -3 and -4, five on chromosome-2, four on chromosome-5, and two on chromosome-7. The locus ‘Chr06-44248544’ was associated with six traits viz., leucine, isoleucine, alanine, phenylalanine, valine and proline (Fig. 3b). The locus ‘Chr06-42701420’ with five traits viz., threonine, tyrosine, alanine, phenylalanine and proline. Moreover, the loci ‘Chr01-94312547’ and ‘Chr06-148593113’ were associated with four traits each (“histidine, aspartic acid, cysteine, glutamic acid” and “threonine, alanine, phenylalanine, proline”, respectively).

### Identification of candidate genes

We investigated a putative gene positioned inside the linked region, which showed a strong association with the trait of interest. The significant SNPs identified during the GWAS were properly placed on their respective chromosomes of *Pennisetum glaucum* using the reference genome assembly reported by Ramu et al. (2023). We identified a total of 286 genes that surrounded significant SNPs using the GFF file of the Tift reference genome (Tift23D2B1-P1-P5) (Online Resource S8). Thirty-three genes were found near strongly associated SNP markers (Table 1). One SNP (Chr4-67,844,255) was found within the candidate gene. A BLAST search of the NCBI database revealed several matches, indicating a diverse set of potential genes involved in the amino acid biosynthesis pathway, seed vigour, defence against biotic/abiotic stress factors, growth, development, and control of secondary metabolites. Among these, 16 genes shared similarities with amino acid biosynthesis pathway-related genes.

**Table 1:** List of potential genes located near SNP markers strongly linked with protein and amino acid content in pearl millet

Sr No.	Trait	SNP	*P* value	Gene	Gene locus	Description
1	Asp	Chr01-94312547	7.52E-10	dpca1g031210.840	94,314,379–94,315,983	Cytochrome P450 3A30-like
2	Lys	Chr01-90166787	2.34E-10	dpca1g030220.840	90,161,030–90164779	Protein containing Pre-SET, SAD_SRA, SET domains
3	Lys	Chr01-94231924	1.76E-14	dpca1g031190.840	94,220,623–94220953	Protein containing ABC_tran_CTD, Snapin_Pallidin, Syntaxin_2 domains
4	Arg	Chr01-241192934	7.60E-05	dpca1g062110.840	241,179,867–241,181,270	Indole-3-acetatebeta-glucosyltransferaseXnoC3
5	Lys	Chr02-121838429	5.97E-17	dpca2g137280.840	121,808,834–121812262	Protein that binds to Fpr1p (FKBP12)
6	Trp	Chr02-249286963	6.79E-05	dpca2g164250.840	249,310,472–249311095	ProteincontainingGamma-thionin,SLR1-BPdomains
7	Arg	Chr02-37483579	1.67E-05	dpca2g106660.840	37,536,478–37,538,856	UDP-glucose6-dehydrogenase
8	Tyr	Chr2-66,144,770	7.22E-05	dpca2g118060.840	66,138,582–66,144,612	Inorganicpyrophosphatase
9	Arg	Chr03-152794146	3.00E-07	dpca3g213710.840	152,636,217–152,637,071	Description not available
10	Trp	Chr03-72850957	2.12E-09	dpca3g199620.840	72,684,412–72,686,330	Uncharacterized protein
11	Asp	Chr03-239767386	3.37E-05	dpca3g231370.840	239,802,803–239804233	3-ketoacyl-CoAsynthase1ProteincontainingACP_syn_III
12	Ser	Chr03-159973304	2.74E-05	dpca3g214820.840	159,915,407–159918711	ProteincontainingKdo,Pkinase,Pkinase_Tyrdomains
13	Met	Chr3-36,627,361	2.46E-05	dpca3g188670.840	36,539,087–36541608	sn-glycerol-3-phosphatetransporter
14	Lys	Chr3-60,817,768	7.45E-05	dpca3g195760.840	60,811,391–60814819	ProteincontainingPkinase,Pkinase_Tyrdomains
15	Asp	Chr3-237,540,103	6.46E-05	dpca3g230850.840	237,486,622–237,492,419	ProteincontainingPkinase,Pkinase_Tyrdomains
16	Cys	Chr3-239,833,532	2.22E-05	dpca3g231370.840	239,802,803–239804233	3-ketoacyl-CoAsynthase1
17	Met	Chr3-243,699,167	4.39E-06	dpca3g232280.840	243,683,647–243,686,034	SJCHGC08170protein(Fragment)
18	Met	Chr04-6908911	2.47E-07	dpca4g264760.840	6,917,313–6,919,816	Protein containing Kinase-like, NAF, Pkinase, Pkinase_Tyr domains
19	Trp	Chr4-67,844,255	6.11E-05	dpca4g286940.840	67,843,921–67,845,301	S-norcoclaurine synthase 1-like
20	Met	Chr05-28823300	1.67E-07	dpca5g334640.840	28,477,703–28478182	Description not available
21	Trp	Chr05-56191227	4.47E-08	dpca5g339610.840	56,208,261–56208550	Protein containing KCNQC3-Ank-G_bd domains
22	Ser	Chr5-106,999,828	5.67E-06	dpca5g357640.840	106,992,536–106999671	3-deoxy-7-phosphoheptulonate synthase
23	Trp	Chr5-130,400,756	6.79E-05	dpca5g365800.840	130,400,990–130404772	Cystathionine gamma-synthase
24	Asp	Chr5-131,237,639	4.58E-06	dpca5g366140.840	131,242,926–131,243,532	Serine/threonine-protein kinase STY8-like isoform X3
25	Ser	Chr5-106,999,828	5.67E-06	dpca5g357640.840	106,992,536–106999671	3-deoxy-7-phosphoheptulonate synthase
26	Val	Chr5-77,862,672	5.74E-05	dpca5g345960.840	77,863,061–77866077	Phosphoglycerate kinase Protein containing PGK domains
27	Ser	Chr06-147646969	9.82E-07	dpca6g430640.840	147,615,887–147,618,268	Kinesin-like protein KIN12B
28	Ser	Chr06-154131992	4.71E-07	dpca6g431590.840	154,157,956–154,158,315	Description not available
29	Trp	Chr06-249278794	1.96E-13	dpca6g450910.840	249,278,938–249,279,165	Uncharacterized protein
30	Lys	Chr06-172222184	1.22E-15	dpca6g434460.840	172,230,901–172231461	Uncharacterized protein
31	Ala	Chr6-151,456,453	4.66E-05	dpca6g431180.840	151,441,063–151442479	Ribose-5-phosphate isomerase B
32	Arg	Chr6-155,762,384	7.68E-05	dpca6g431780.840	155,769,447–155,770,910	3-ketoacyl-CoA synthase 1
33	Lys	Chr07-196098235	1.91E-12	dpca7g517900.840	196,124,589–196,125,532	Uncharacterized protein

Functional annotation revealed that these genes contained molecular active sites important for controlling amino acid biosynthesis. Ribose-5-phosphate isomerase (dpca6g431180.840), a protein with Cupin_2, Cupin_7, and LacAB_rpiB domains, plays a role in alanine biosynthesis from ribose. It catalyses the isomerization of D-ribulose 5-phosphate to D-ribose 5-phosphate and increases erythrose 4-phosphate synthesis. As a result, erythrose 4-phosphate is implicated in the synthesis of numerous amino acids, including alanine. This implies that these genes may function as cofactors in these pathways or control genes via diverse chemical pathways to improve the synthesis of various essential and non-essential amino acids.

Moreover, certain candidate genes were directly associated with antioxidant biosynthetic pathways across all SNP datasets, including 3-deoxy-7-phosphoheptulonate synthase (dpca5g357640.840), protein containing DAHP_synth_2, Pkinase, and Pkinase_Tyr domains involved in the synthesis of the first of the biosynthetic pathways for the aromatic amino acids phenylalanine, tryptophan, and tyrosine from phosphoenolpyruvate. Kinesin-like protein KIN12B (dpca6g430640.840), FKBP12 (dpca2g137280.840), ABC_tran_CTD, Snapin_Pallidin, Syntaxin_2 domains (dpca1g031190.840), and Pre-SET, SAD_SRA, SET domainspurple acid phosphatase 15 (dpca1g030220.840) were found to be the most significant (*P* = 9.81756E-07 to 5.96773E-17). In future studies, these selected candidate genes will be validated further via haplotyping and functional characterisation.

## Discussion

Hidden hunger and malnutrition are serious threats to human health, especially in Asian and African countries, where the majority of people rely on vegetarian sources of nutrition. Especially, protein and essential amino acid contents are the major concern in vegetarian diets (Shewry 2007). Genetic improvement of quantity and quality of protein in major cereals like pearl millet are therefore important in tackling the problem of hidden hunger worldwide. The genomic regions and genetic basis of protein and different amino acids have been successfully explored previously in several crops like sorghum (Kimani et al. 2020), wheat (Peng et al. 2018; Nigro et al. 2019), rice (He et al. 2022; Wang et al. 2023) and maize (Deng et al. 2017; Zheng et al. 2021). Though pearl millet is one of the major staple foods in Asian and African countries and ranked fifth cereal worldwide (Singh and Gupta 2019), no efforts have yet been made to identify the genomic regions associated with protein and amino acid content utilising its germplasm resource. In this present study, a large and continuous phenotypic variation was observed for all the traits under study including protein content as well as essential and non-essential amino acids, which indicated the large allelic diversity in the PMiGAP and these characteristics are quantitative in nature.

At the molecular level, population structure and principal component analysis confirmed the presence of high diversity in the PMiGAP panel. Further, the availability of the new pan-genomic set of 435 K SNPs used in the study offered a higher resolution in association analysis (Ramu et al. 2023). The higher molecular diversity of PMiGAP was reported earlier in many studies with different sets of SNPs (Sehgal et al. 2015). The LD decay in the population of 161 genotypes we studied remained at *r*^2^ = 0.2 at 107 kb, which is quite higher than the previously reported studies in pearl millet with 994 accessions (Varshney et al. 2017), comparable to another study that included 398 accessions (Serba et al. 2019), but lower than reported in sorghum (Kimani et al. 2020), wheat and rice (Wang et al. 2023). The number of SNPs we used in the current study was much higher than the suggested LD decay.

The higher number of pleiotropic loci recorded in the present study could explain the high correlation observed among the amino acids. No pleiotropic locus was detected for protein with amino acids, which indicated that the protein content is a complex trait and the genomic regions controlling it may lie beyond those of the amino acids. A total of 286 genes were identified around the significant SNPs associated with different amino acid synthesis pathways. Sixteen candidate genes, including glycerol-3-phosphate transporter protein with MFS_1 domains, 3-ketoacyl-CoA synthase1, 3-deoxy-7-phosphoheptulonate synthase, serine/threonine-protein kinase, and ribose-5-phosphate isomerase, were found to be directly associated with the amino acid biosynthesis pathway across all selected SNP data sets. These candidates have been identified near the SNPs revealed in this study, but they will require further validation via gene editing, RNAi, VIGS methods in future studies.

The identification of significant associated alleles will determine their roles in amino acid biosynthesis, ensuring their effective application in crop development projects. Similar approaches, as we report here, were also adopted by Yadav et al. (2021) for association analysis with variation in antioxidant content and they reported that 218 SNPs were strongly associated with antioxidant taris (DPPH and FRAP activity) at high confidence [–log (*P*) > 3.0–7.4]. Furthermore, flanking regions of significantly associated SNPs revealed that 18 candidate genes related to antioxidant pathway genes (flavanone 7-O-beta-glycosyltransferase, GDSL esterase/lipase, glutathione S-transferase) residing within or near the association signal that can be selected for further functional characterization.

Similarly, 47 significant SNPs were associated with tocopherol and tocotrienol content, identifying eighty-eight genes predicted to be transcription factors, including NAC domain transcript factors and Myb domain transcript (Luo et al. 2020). However, they chose EgHGGT (homogentisate geranylgeranyl transferase) for further investigation since it is involved in the production of tocotrienols and has higher expression levels in the mesocarp than in other tissues. Many researchers have previously sought to identify antioxidant-associated candidate genes in maize and other cereal crops. Saïdou et al. (2014) conducted association mapping analyses for key agronomic parameters in pearl millet. Association analysis in maize marked SNPs linked with phenotypic variation of vitamin E and discovered an indel 85 Kb upstream of the ZmVTE4 gene, which was revealed to play a role in the expression of the ZmVTE4 gene (Li et al. 2012). Gangasetty and coworkers (2023) reported substantial allelic associations for grain iron, zinc, and other agronomic characteristics in pearl millet.

## Conclusion

The current study used GWAS to identify genetic loci associated with protein and amino acids in pearl millet germplasm. The germplasm population (PMiGAP) used demonstrated a wide range of variability in protein and amino acid contents and identified 23 highly significant SNP markers (– log_10_*P* ≥ 5.5) and 544 significant SNP markers (– log_10_*P* ≥ 4) linked with protein and amino acid content. These findings for the first time have dissected the genetic architecture of protein and amino acids in pearl millet germplasm panel for use in pearl millet breeding programmes. The study identified 33 promising candidate genes, which, once validated, can be a valuable resource for selecting these nutritional traits in pearl millet breeding schemes to combat hidden hunger worldwide. Apart from protein and amino acids, the PMiGAP we used, has been studied previously for several other nutritionally important traits such as starch, antioxidants, and other healthful traits, providing opportunities to simultaneously combine findings of this and of the other previously reported studies to combine various traits in future pearl millet varieties.

### Supplementary Information

Below is the link to the electronic supplementary material.

Supplementary file1 (PDF 365 KB)

Supplementary file2 (PDF 671 KB)

Supplementary file3 (PDF 287 KB)

Supplementary file4 (PDF 266 KB)

Supplementary file5 (PDF 721 KB)

Supplementary file6 (PDF 730 KB)

Supplementary file7 (PDF 726 KB)

Supplementary file8 (PDF 487 KB)

## Data Availability

The data supporting the findings of this study are available within the article and its supplementary materials.

## Abbreviations

GWAS: Genome-Wide Association Study
LD: Linkage Disequilibrium
PMiGAP: Pearl Millet Inbred Germplasm Association Panel
SNP: Single Nucleotide Polymorphism
