# Supplementary material for: Genome-wide SNPs and candidate genes underlying the genetic variations for protein and amino acids in pearl millet (Pennisetum glaucum) germplasm
Source: Planta. 2024 Jul 27;260(3):63. doi: 10.1007/s00425-024-04495-y (PMC11283402; doi:10.1007/s00425-024-04495-y)
Supplement: Supplementary file 1 — Supplementary file1 (PDF 365 KB) [file 425_2024_4495_MOESM1_ESM.pdf]

Genome-wide SNPs and candidate genes underlying the genetic variations for protein and amino acids in pearl millet (*Pennisetum glaucum*) germplasm

PLANTA

Satbeer Singh[1,2], Chandra Bhan Yadav[1,3], Nelson Lubanga[1], Matthew Hegarty[1], Rattan S. Yadav[1]*

[1] Institute of Biological Environmental and Rural Sciences (IBERS), Aberystwyth University, Aberystwyth, SY23 3EE, United Kingdom

[2] Division of Agrotechnology, Council of Scientific and Industrial Research (CSIR) - Institute of Himalayan Bioresource Technology, Palampur, Himachal Pradesh 176 061, India

[3] Department of Genetics, Genomics, and Breeding, NIAB-EMR, East Malling, ME19 6BJ, United Kingdom

* Corresponding author: rsy@aber.ac.uk

Online Resource S1 Details of PMiGAP genotypes used in study

Sr No.	Accession identifier	PMiGAP Id	Biological status	Country Source
1.	IP-2058	PMiGAP128	Improved cultivar	Nigeria
2.	IP-3098	PMiGAP081	Landrace	India
3.	IP-3110	PMiGAP307	Landrace	India
4.	IP-3122	PMiGAP137	Landrace	India
5.	IP-3125	PMiGAP012	Landrace	India
6.	IP-3175	PMiGAP058	Landrace	India
7.	IP-3389	PMiGAP207	Landrace	India
8.	IP-3564	PMiGAP119	Landrace	India
9.	IP-3593	PMiGAP171	Landrace	India
10.	IP-3636	PMiGAP148	Landrace	India
11.	IP-3732	PMiGAP168	Landrace	India
12.	IP-3757	PMiGAP041	Landrace	India
13.	IP-3865	PMiGAP098	Landrace	India
14.	IP-3890	PMiGAP063	Landrace	India
15.	IP-4020	PMiGAP016	Landrace	India
16.	IP-4378	PMiGAP311	Landrace	India
17.	IP-4927	PMiGAP257	Breeding line	Senegal
18.	IP-4952	PMiGAP244	Landrace	Uganda
19.	IP-4974	PMiGAP179	Landrace	Nigeria
20.	IP-5031	PMiGAP266	Landrace	Nigeria
21.	IP-5131	PMiGAP222	Landrace	Niger
22.	IP-5272	PMiGAP157	Landrace	Niger
23.	IP-5438	PMiGAP332	Landrace	Niger
24.	IP-5441	PMiGAP177	Landrace	Niger
25.	IP-5695	PMiGAP104	Breeding line	Nigeria
26.	IP-5713	PMiGAP140	Landrace	Nigeria
27.	IP-5816	PMiGAP249	Breeding line	Senegal
28.	IP-5900	PMiGAP306	Landrace	Senegal
29.	IP-5923	PMiGAP226	Breeding line	Senegal
30.	IP-6037	PMiGAP136	Landrace	Central African Republic
31.	IP-6060	PMiGAP049	Breeding line	Central African Republic
32.	IP-6098	PMiGAP323	Landrace	Niger
33.	IP-6099	PMiGAP149	Landrace	Niger
34.	IP-6103	PMiGAP344	Breeding line	Niger
35.	IP-6111	PMiGAP122	Landrace	Niger
36.	IP-6112	PMiGAP042	Landrace	Niger
37.	IP-6146	PMiGAP138	Landrace	Cameroon
38.	IP-6584	PMiGAP013	-	Malawi
39.	IP-6769	PMiGAP021	Landrace	Malawi
40.	IP-6882	PMiGAP247	Landrace	Kenya
41.	IP-6891	PMiGAP174	Landrace	Kenya
42.	IP-6892	PMiGAP314	Landrace	Kenya

Sr No.	Accession identifier	PMiGAP Id	Biological status	Country Source
43.	IP-7470	PMiGAP183	Landrace	Tanzania
44.	IP-7536	PMiGAP296	Landrace	India
45.	IP-7633	PMiGAP047	Landrace	India
46.	IP-7910	PMiGAP166	Breeding line	Niger
47.	IP-7922	PMiGAP236	Landrace	ICRISAT
48.	IP-7942	PMiGAP073	Landrace	ICRISAT
49.	IP-7952	PMiGAP176	Landrace	ICRISAT
50.	IP-7953	PMiGAP213	Landrace	ICRISAT
51.	IP-7967	PMiGAP200	Breeding line	ICRISAT
52.	IP-8002	PMiGAP151	Landrace	Sudan
53.	IP-8129	PMiGAP218	Landrace	ICRISAT
54.	IP-8166	PMiGAP258	Landrace	ICRISAT
55.	IP-8172	PMiGAP201	Landrace	ICRISAT
56.	IP-8174	PMiGAP346	Breeding/Research material	ICRISAT
57.	IP-8181	PMiGAP091	Landrace	ICRISAT
58.	IP-8182	PMiGAP302	Landrace	ICRISAT
59.	IP-8187	PMiGAP227	Landrace	ICRISAT
60.	IP-8210	PMiGAP167	Landrace	ICRISAT
61.	IP-8276	PMiGAP050	Breeding line	ICRISAT
62.	IP-8280	PMiGAP114	Landrace	ICRISAT
63.	IP-8294	PMiGAP279	Landrace	ICRISAT
64.	IP-8426	PMiGAP181	Landrace	Nigeria
65.	IP-8761	PMiGAP109	Landrace	Botswana
66.	IP-8767	PMiGAP008	Landrace	Botswana
67.	IP-8786	PMiGAP312	Landrace	Botswana
68.	IP-8863	PMiGAP262	Landrace	Zambia
69.	IP-8949	PMiGAP090	Breeding line	Togo
70.	IP-8972	PMiGAP180	Landrace	Togo
71.	IP-9242	PMiGAP134	Landrace	Mali
72.	IP-9301	PMiGAP139	Landrace	Togo
73.	IP-9347	PMiGAP056	Improved cultivar	Ghana
74.	IP-9351	PMiGAP132	Landrace	Ghana
75.	IP-9407	PMiGAP076	Landrace	Ghana
76.	IP-9496	PMiGAP087	Landrace	Ghana
77.	IP-9651	PMiGAP233	Landrace	Nigeria
78.	IP-9692	PMiGAP232	Landrace	Nigeria
79.	IP-9710	PMiGAP135	Landrace	Nigeria
80.	IP-9840	PMiGAP080	Landrace	Sudan
81.	IP-9854	PMiGAP147	Landrace	Sudan
82.	IP-9969	PMiGAP246	Landrace	Zambia
83.	IP-10085	PMiGAP159	Landrace	Mali
84.	IP-10343	PMiGAP093	Breeding line	Nigeria
85.	IP-10471	PMiGAP219	Landrace	Zimbabwe
86.	IP-10486	PMiGAP099	Landrace	Zimbabwe
87.	IP-10488	PMiGAP069	Landrace	Zimbabwe

Sr No.	Accession identifier	PMiGAP Id	Biological status	Country Source
88.	IP-10539	PMiGAP133	Landrace	Senegal
89.	IP-10701	PMiGAP215	Landrace	Mali
90.	IP-10705	PMiGAP025	Improved cultivar	Mali
91.	IP-10759	PMiGAP336	Landrace	Sudan
92.	IP-10811	PMiGAP033	Landrace	Sudan
93.	IP-10820	PMiGAP088	Landrace	Sudan
94.	IP-10953	PMiGAP141	Breeding line	Kenya
95.	IP-10964	PMiGAP084	Landrace	Kenya
96.	IP-11310	PMiGAP152	-	Burkina Faso
97.	IP-11311	PMiGAP075	Landrace	Burkina Faso
98.	IP-11346	PMiGAP264	Landrace	Burkina Faso
99.	IP-11358	PMiGAP267	Landrace	Burkina Faso
100.	IP-11577	PMiGAP035	Improved cultivar	Burkina Faso
101.	IP-11584	PMiGAP182	Landrace	Burkina Faso
102.	IP-11593	PMiGAP220	Improved cultivar	Burkina Faso
103.	IP-11670	PMiGAP261	Landrace	United Kingdom
104.	IP-11677	PMiGAP064	Landrace	Sudan
105.	IP-11763	PMiGAP329	Landrace	South Africa
106.	IP-11765	PMiGAP155	Landrace	South Africa
107.	IP-11984	PMiGAP065	Landrace	Nigeria
108.	IP-12116	PMiGAP285	Landrace	Nigeria
109.	IP-12128	PMiGAP317	Landrace	Nigeria
110.	IP-12298	PMiGAP009	Breeding line	Nigeria
111.	IP-12322	PMiGAP117	Landrace	Nigeria
112.	IP-12364	PMiGAP320	Landrace	Nigeria
113.	IP-12840	PMiGAP059	Landrace	Botswana
114.	IP-12925	PMiGAP067	Landrace	Ghana
115.	IP-12967	PMiGAP223	Improved cultivar	Malawi
116.	IP-13016	PMiGAP096	Breeding line	Mali
117.	IP-13149	PMiGAP071	Landrace	Niger
118.	IP-13154	PMiGAP077	Landrace	Niger
119.	IP-13180	PMiGAP205	Landrace	Nigeria
120.	IP-13324	PMiGAP281	Landrace	Sudan
121.	IP-13344	PMiGAP202	Improved cultivar	Sudan
122.	IP-13363	PMiGAP283	Landrace	Tanzania
123.	IP-13459	PMiGAP298	Landrace	India
124.	IP-13817	PMiGAP217	Breeding line	Burkina Faso
125.	IP-13964	PMiGAP089	Landrace	Zimbabwe
126.	IP-13971	PMiGAP011	Landrace	Zimbabwe
127.	IP-14210	PMiGAP340	Landrace	Cameroon
128.	IP-14624	PMiGAP331	Landrace	Cameroon
129.	IP-14849	PMiGAP305	Landrace	Cameroon
130.	IP-15320	PMiGAP124	Landrace	India
131.	IP-15533	PMiGAP072	Breeding line	Burkina Faso
132.	IP-15553	PMiGAP273	Landrace	Burkina Faso

Sr No.	Accession identifier	PMiGAP Id	Biological status	Country Source
133.	IP-15872	PMiGAP229	Improved cultivar	Tanzania
134.	IP-15917	PMiGAP007	Landrace	Togo
135.	IP-16096	PMiGAP046	Landrace	India
136.	IP-16120	PMiGAP290	Landrace	India
137.	IP-16289	PMiGAP186	Landrace	Zimbabwe
138.	IP-16403	PMiGAP126	Landrace	Zimbabwe
139.	IP-17028	PMiGAP339	Landrace	Zimbabwe
140.	IP-17099	PMiGAP269	Landrace	Zimbabwe
141.	IP-17150	PMiGAP303	Landrace	Zimbabwe
142.	IP-17554	PMiGAP079	Landrace	Togo
143.	IP-17611	PMiGAP198	Landrace	Togo
144.	IP-17690	PMiGAP066	Landrace	Togo
145.	IP-18062	PMiGAP020	Landrace	Pakistan
146.	IP-18147	PMiGAP038	Breeding line	Pakistan
147.	IP-18168	PMiGAP271	Landrace	Mali
148.	IP-18246	PMiGAP272	Landrace	India
149.	IP-18293	PMiGAP204	Landrace	ICRISAT
150.	IP-18500	PMiGAP263	Landrace	Namibia
151.	IP-18621	PMiGAP338	Landrace	Namibia
152.	IP-19334	PMiGAP308	Landrace	Namibia
153.	IP-19361	PMiGAP313	Breeding line	Namibia
154.	IP-19386	PMiGAP060	Improved cultivar	Namibia
155.	IP-19388	PMiGAP078	Landrace	Namibia
156.	IP-19612	PMiGAP214	Landrace	Niger
157.	IP-19613	PMiGAP150	Landrace	Niger
158.	IP-19626	PMiGAP118	Landrace	Niger
159.	IP-21020	PMiGAP173	Landrace	Nigeria
160.	IP-21169	PMiGAP210	Improved cultivar	ICRISAT
161.	IP-21206	PMiGAP144	Breeding line	ICRISAT
