# Supplementary material for: Genome-wide SNPs and candidate genes underlying the genetic variations for protein and amino acids in pearl millet (Pennisetum glaucum) germplasm
Source: Planta. 2024 Jul 27;260(3):63. doi: 10.1007/s00425-024-04495-y (PMC11283402; doi:10.1007/s00425-024-04495-y)
Supplement: Supplementary file 2 — Supplementary file2 (PDF 671 KB) [file 425_2024_4495_MOESM2_ESM.pdf]

Genome-wide SNPs and candidate genes underlying the genetic variations for protein and amino acids in pearl millet (*Pennisetum glaucum*) germplasm

PLANTA

Satbeer Singh[1,2], Chandra Bhan Yadav[1,3], Nelson Lubanga[1], Matthew Hegarty[1], Rattan S. Yadav[1]*

[1] Institute of Biological Environmental and Rural Sciences (IBERS), Aberystwyth University, Aberystwyth, SY23 3EE, United Kingdom

[2] Division of Agrotechnology, Council of Scientific and Industrial Research (CSIR) - Institute of Himalayan Bioresource Technology, Palampur, Himachal Pradesh 176 061, India

[3] Department of Genetics, Genomics, and Breeding, NIAB-EMR, East Malling, ME19 6BJ, United Kingdom

* Corresponding author: rsy@aber.ac.uk

Online Resource S2 r2-value for the calibration curves for all measured amino acids at concentrations between 1.82 and 750 µM (0.43 and 16.2 µM for Trp)

Amino Acid	r2	Equation
**Asp**	0.999	Y = 1.0430*X - 1.0317
**Ser**	0.999	Y = 1.0550*X - 1.0976
**His**	0.996	Y = 1.0282*X - 1.083
**Thr**	0.987	Y = 0.9567*X - 1.0469
**Pro**	0.995	Y = 0.9869*X - 1.1408
**Val**	0.999	Y = 0.9476*X - 1.004
**Cys**	0.999	Y = 0.9959*X - 0.9926
**Leu**	0.999	Y = 0.9189*X - 1.0044
**Glu**	0.999	Y = 1.0172*X - 1.0398
**Gly**	0.997	Y = 1.0710*X - 1.1026

Amino Acid	r2	Equation
**Arg**	0.999	Y = 1.0124*X - 1.0966
**Ala**	0.999	Y = 0.9700*X - 1.0327
**Tyr**	0.992	Y = 0.9839*X - 1.1124
**Met**	0.998	Y = 0.9450*X - 1.0218
**Ile**	0.998	Y = 0.9577*X - 1.0191
**Phe**	0.995	Y = 1.0555*X - 1.1098
**Lys**	0.995	Y = 0.9099*X - 1.281
**Trp**	1.000	Y = 1.0102*X - 5.5315
