# Supplementary material for: Genome-wide SNPs and candidate genes underlying the genetic variations for protein and amino acids in pearl millet (Pennisetum glaucum) germplasm
Source: Planta. 2024 Jul 27;260(3):63. doi: 10.1007/s00425-024-04495-y (PMC11283402; doi:10.1007/s00425-024-04495-y)
Supplement: Supplementary file 4 — Supplementary file4 (PDF 266 KB) [file 425_2024_4495_MOESM4_ESM.pdf]

Genome-wide SNPs and candidate genes underlying the genetic variations for protein and amino acids in pearl millet (*Pennisetum glaucum*) germplasm

PLANTA

Satbeer Singh[1,2], Chandra Bhan Yadav[1,3], Nelson Lubanga[1], Matthew Hegarty[1], Rattan S. Yadav[1]*

[1] Institute of Biological Environmental and Rural Sciences (IBERS), Aberystwyth University, Aberystwyth, SY23 3EE, United Kingdom

[2] Division of Agrotechnology, Council of Scientific and Industrial Research (CSIR) - Institute of Himalayan Bioresource Technology, Palampur, Himachal Pradesh 176 061, India

[3] Department of Genetics, Genomics, and Breeding, NIAB-EMR, East Malling, ME19 6BJ, United Kingdom

* Corresponding author: rsy@aber.ac.uk

Online Resource S4 Marker distribution and density

Chromosome	Number of SNPs	Size covered (Mb)	Density (SNPs/Mb)
Chromosome 1	62819	298	210
Chromosome 2	78109	269	290
Chromosome 3	71230	325	219
Chromosome 4	54956	240	229
Chromosome 5	52430	170	309
Chromosome 6	67101	281	239
Chromosome 7	48735	275	177
Average per chr.	62197	266	239
Total over all chr.	435380	1859	
