# Supplementary material for: Genome-wide SNPs and candidate genes underlying the genetic variations for protein and amino acids in pearl millet (Pennisetum glaucum) germplasm
Source: Planta. 2024 Jul 27;260(3):63. doi: 10.1007/s00425-024-04495-y (PMC11283402; doi:10.1007/s00425-024-04495-y)
Supplement: Supplementary file 6 — Supplementary file6 (PDF 730 KB) [file 425_2024_4495_MOESM6_ESM.pdf]

Genome-wide SNPs and candidate genes underlying the genetic variations for protein and amino acids in pearl millet (*Pennisetum glaucum*) germplasm

PLANTA

Satbeer Singh[1,2], Chandra Bhan Yadav[1,3], Nelson Lubanga[1], Matthew Hegarty[1], Rattan S. Yadav[1]*

[1] Institute of Biological Environmental and Rural Sciences (IBERS), Aberystwyth University, Aberystwyth, SY23 3EE, United Kingdom

[2] Division of Agrotechnology, Council of Scientific and Industrial Research (CSIR) - Institute of Himalayan Bioresource Technology, Palampur, Himachal Pradesh 176 061, India

[3] Department of Genetics, Genomics, and Breeding, NIAB-EMR, East Malling, ME19 6BJ, United Kingdom

* Corresponding author: rsy@aber.ac.uk

Online Resource S6 List of significant Marker Associations at *P*-value<0.0001 ($-\log_{10}P=4$)

Sr No	Trait	SNP	Chr	Position	P.value	-log10
1	Ala	Chr02-260587678	2	260587678	6.5E-05	4.2
2	Ala	Chr03-63998406	3	63998406	7.86E-05	4.1
3	Ala	Chr04-44286610	4	44286610	5.08E-05	4.3
4	Ala	Chr05-56381500	5	56381500	2.21E-05	4.7
5	Ala	Chr05-84850820	5	84850820	2.52E-05	4.6
6	Ala	Chr05-140112743	5	140112743	6.43E-05	4.2
7	Ala	Chr06-42701420	6	42701420	5.35E-06	5.3
8	Ala	Chr06-42720239	6	42720239	3.02E-05	4.5
9	Ala	Chr06-42852393	6	42852393	5.4E-05	4.3
10	Ala	Chr06-43329491	6	43329491	5.57E-05	4.3
11	Ala	Chr06-43671756	6	43671756	5.01E-05	4.3
12	Ala	Chr06-44248544	6	44248544	6.55E-06	5.2
13	Ala	Chr06-148161712	6	148161712	5.71E-05	4.2
14	Ala	Chr06-148593113	6	148593113	1.51E-05	4.8
15	Ala	Chr06-151456453	6	151456453	4.66E-05	4.3
16	Arg	Chr01-90203370	1	90203370	4.88E-05	4.3
17	Arg	Chr01-226869405	1	226869405	5.8E-05	4.2
18	Arg	Chr01-241192934	1	241192934	7.6E-05	4.1
19	Arg	Chr01-261635663	1	261635663	7.46E-05	4.1
20	Arg	Chr01-272773528	1	272773528	1.94E-05	4.7
21	Arg	Chr01-272828045	1	272828045	2.07E-05	4.7
22	Arg	Chr02-2669720	2	2669720	1.15E-05	4.9
23	Arg	Chr02-37441685	2	37441685	5.99E-05	4.2
24	Arg	Chr02-37441686	2	37441686	5.99E-05	4.2
25	Arg	Chr02-37448771	2	37448771	5.61E-05	4.3
26	Arg	Chr02-37454027	2	37454027	6.46E-05	4.2
27	Arg	Chr02-37454045	2	37454045	5.1E-05	4.3
28	Arg	Chr02-37457222	2	37457222	2.09E-05	4.7
29	Arg	Chr02-37483579	2	37483579	1.67E-05	4.8
30	Arg	Chr02-253101995	2	253101995	2.48E-05	4.6
31	Arg	Chr03-149476119	3	149476119	5.2E-05	4.3
32	Arg	Chr03-152794146	3	152794146	3E-07	6.5
33	Arg	Chr03-155597939	3	155597939	1.84E-05	4.7
34	Arg	Chr03-159905183	3	159905183	1.75E-05	4.8
35	Arg	Chr04-47125957	4	47125957	5.38E-05	4.3
36	Arg	Chr04-48575150	4	48575150	1.75E-05	4.8
37	Arg	Chr04-185236123	4	185236123	2.84E-05	4.5
38	Arg	Chr05-87250676	5	87250676	7.86E-05	4.1
39	Arg	Chr06-154131992	6	154131992	4.09E-05	4.4
40	Arg	Chr06-154333801	6	154333801	1.39E-05	4.9
41	Arg	Chr06-154972950	6	154972950	6.71E-05	4.2
42	Arg	Chr06-155762384	6	155762384	7.68E-05	4.1
43	Arg	Chr06-252992569	6	252992569	5.4E-05	4.3
44	Arg	Chr06-264330868	6	264330868	4.4E-06	5.4
45	Arg	Chr06-277493247	6	277493247	5.52E-05	4.3

Sr No	Trait	SNP	Chr	Position	P.value	-log10
46	Arg	Chr07-134016112	7	134016112	7.29E-05	4.1
47	Arg	Chr07-171605023	7	171605023	5.92E-06	5.2
48	Arg	Chr07-207576451	7	207576451	6.76E-05	4.2
49	Arg	Chr07-207578128	7	207578128	4.87E-05	4.3
50	Arg	Chr07-207686820	7	207686820	5.14E-05	4.3
51	Arg	Chr07-207694797	7	207694797	4.87E-05	4.3
52	Arg	Chr07-207696790	7	207696790	4.87E-05	4.3
53	Arg	Chr07-207698929	7	207698929	5.83E-05	4.2
54	Arg	Chr07-207699230	7	207699230	5.3E-05	4.3
55	Asp	Chr01-55371493	1	55371493	1.63E-05	4.8
56	Asp	Chr01-55371752	1	55371752	3.46E-05	4.5
57	Asp	Chr01-90167351	1	90167351	1.95E-05	4.7
58	Asp	Chr01-94312547	1	94312547	7.52E-10	9.1
59	Asp	Chr01-185139326	1	185139326	2.61E-05	4.6
60	Asp	Chr01-217678759	1	217678759	2.74E-06	5.6
61	Asp	Chr01-298411919	1	298411919	9.56E-06	5.0
62	Asp	Chr02-43578448	2	43578448	4.69E-05	4.3
63	Asp	Chr02-93450528	2	93450528	2.28E-05	4.6
64	Asp	Chr02-93501059	2	93501059	3.84E-05	4.4
65	Asp	Chr02-93541206	2	93541206	3.84E-05	4.4
66	Asp	Chr02-118163944	2	118163944	6.24E-05	4.2
67	Asp	Chr02-118410674	2	118410674	6.24E-05	4.2
68	Asp	Chr02-156304901	2	156304901	4.41E-05	4.4
69	Asp	Chr02-193359922	2	193359922	3.31E-05	4.5
70	Asp	Chr02-195974730	2	195974730	3.28E-05	4.5
71	Asp	Chr02-196670206	2	196670206	4.5E-05	4.3
72	Asp	Chr02-209374964	2	209374964	5.43E-05	4.3
73	Asp	Chr02-265087146	2	265087146	1.46E-05	4.8
74	Asp	Chr03-198193118	3	198193118	7.57E-05	4.1
75	Asp	Chr03-237540103	3	237540103	6.46E-05	4.2
76	Asp	Chr03-237566752	3	237566752	3.07E-06	5.5
77	Asp	Chr03-237710932	3	237710932	5E-05	4.3
78	Asp	Chr03-238142136	3	238142136	1.29E-05	4.9
79	Asp	Chr03-238162581	3	238162581	7.83E-05	4.1
80	Asp	Chr03-239564544	3	239564544	5.77E-06	5.2
81	Asp	Chr03-239762570	3	239762570	5.42E-05	4.3
82	Asp	Chr03-239767386	3	239767386	3.37E-05	4.5
83	Asp	Chr04-100745272	4	100745272	3.84E-05	4.4
84	Asp	Chr04-179597069	4	179597069	1.42E-05	4.8
85	Asp	Chr05-121631325	5	121631325	4.16E-05	4.4
86	Asp	Chr05-131169450	5	131169450	7.9E-05	4.1
87	Asp	Chr05-131237639	5	131237639	4.58E-06	5.3
88	Asp	Chr05-159675841	5	159675841	6.43E-05	4.2
89	Asp	Chr06-124109057	6	124109057	4.54E-05	4.3
90	Asp	Chr07-120107479	7	120107479	6.33E-05	4.2
91	Asp	Chr07-158564034	7	158564034	3.06E-05	4.5

Sr No	Trait	SNP	Chr	Position	P.value	-log10
92	Cys	Chr01-64382380	1	64382380	7.02E-05	4.2
93	Cys	Chr01-64427363	1	64427363	6.89E-05	4.2
94	Cys	Chr01-64687910	1	64687910	6.91E-05	4.2
95	Cys	Chr01-92027967	1	92027967	1.23E-05	4.9
96	Cys	Chr01-92248603	1	92248603	3.28E-05	4.5
97	Cys	Chr01-94312547	1	94312547	6.89E-05	4.2
98	Cys	Chr01-107682403	1	107682403	1.73E-05	4.8
99	Cys	Chr01-130249468	1	130249468	1.42E-05	4.8
100	Cys	Chr01-185139326	1	185139326	5.29E-05	4.3
101	Cys	Chr01-195681683	1	195681683	1.25E-05	4.9
102	Cys	Chr01-228026675	1	228026675	7.43E-05	4.1
103	Cys	Chr02-20214476	2	20214476	2.77E-05	4.6
104	Cys	Chr02-39671080	2	39671080	7.74E-05	4.1
105	Cys	Chr02-239573425	2	239573425	1.66E-05	4.8
106	Cys	Chr02-258586012	2	258586012	3.5E-05	4.5
107	Cys	Chr02-260402443	2	260402443	4.96E-05	4.3
108	Cys	Chr02-265086868	2	265086868	2.46E-05	4.6
109	Cys	Chr03-3945755	3	3945755	2.91E-05	4.5
110	Cys	Chr03-4165387	3	4165387	2.43E-05	4.6
111	Cys	Chr03-17635594	3	17635594	5.94E-05	4.2
112	Cys	Chr03-51499160	3	51499160	5E-05	4.3
113	Cys	Chr03-147076298	3	147076298	4.56E-05	4.3
114	Cys	Chr03-183575705	3	183575705	9.41E-06	5.0
115	Cys	Chr03-214113781	3	214113781	4.33E-06	5.4
116	Cys	Chr03-237710932	3	237710932	4.59E-05	4.3
117	Cys	Chr03-238431872	3	238431872	4.38E-05	4.4
118	Cys	Chr03-239100112	3	239100112	1.62E-05	4.8
119	Cys	Chr03-239169662	3	239169662	1.25E-05	4.9
120	Cys	Chr03-239230325	3	239230325	3.19E-05	4.5
121	Cys	Chr03-239335409	3	239335409	4.28E-05	4.4
122	Cys	Chr03-239342534	3	239342534	2.5E-05	4.6
123	Cys	Chr03-239349291	3	239349291	2.22E-05	4.7
124	Cys	Chr03-239361986	3	239361986	1.25E-05	4.9
125	Cys	Chr03-239406600	3	239406600	5.02E-05	4.3
126	Cys	Chr03-239413397	3	239413397	5.02E-05	4.3
127	Cys	Chr03-239439074	3	239439074	2.93E-06	5.5
128	Cys	Chr03-239510790	3	239510790	2.22E-05	4.7
129	Cys	Chr03-239511396	3	239511396	8.69E-06	5.1
130	Cys	Chr03-239511439	3	239511439	3.21E-05	4.5
131	Cys	Chr03-239514663	3	239514663	6.67E-05	4.2
132	Cys	Chr03-239548410	3	239548410	1.47E-05	4.8
133	Cys	Chr03-239567465	3	239567465	3.09E-05	4.5
134	Cys	Chr03-239704367	3	239704367	1.25E-05	4.9
135	Cys	Chr03-239720456	3	239720456	2.22E-05	4.7
136	Cys	Chr03-239833532	3	239833532	2.22E-05	4.7
137	Cys	Chr03-239835699	3	239835699	3.09E-05	4.5

Sr No	Trait	SNP	Chr	Position	P.value	-log10
138	Cys	Chr03-239890863	3	239890863	6.7E-05	4.2
139	Cys	Chr04-204999169	4	204999169	1.13E-05	4.9
140	Cys	Chr05-92008291	5	92008291	1.4E-05	4.9
141	Cys	Chr06-248116663	6	248116663	1.25E-05	4.9
142	Cys	Chr07-1497447	7	1497447	7.49E-05	4.1
143	Cys	Chr07-109320162	7	109320162	3.66E-06	5.4
144	Glu	Chr01-91831977	1	91831977	1.77E-06	5.8
145	Glu	Chr01-91979503	1	91979503	7.5E-06	5.1
146	Glu	Chr01-91979901	1	91979901	2.04E-05	4.7
147	Glu	Chr01-92248603	1	92248603	7.77E-05	4.1
148	Glu	Chr01-93576262	1	93576262	2.16E-05	4.7
149	Glu	Chr01-93619534	1	93619534	6.38E-05	4.2
150	Glu	Chr01-93641283	1	93641283	6.38E-05	4.2
151	Glu	Chr01-93900331	1	93900331	4.19E-05	4.4
152	Glu	Chr01-93900332	1	93900332	4.19E-05	4.4
153	Glu	Chr01-94312547	1	94312547	1.33E-05	4.9
154	Glu	Chr01-119027870	1	119027870	7.11E-05	4.1
155	Glu	Chr01-140766648	1	140766648	6.45E-05	4.2
156	Glu	Chr01-175920885	1	175920885	1.47E-05	4.8
157	Glu	Chr01-185139326	1	185139326	9.96E-06	5.0
158	Glu	Chr01-217678759	1	217678759	4.09E-05	4.4
159	Glu	Chr01-277783844	1	277783844	2.89E-05	4.5
160	Glu	Chr01-283097941	1	283097941	1.95E-05	4.7
161	Glu	Chr01-285641243	1	285641243	7.28E-05	4.1
162	Glu	Chr02-228572807	2	228572807	7.12E-05	4.1
163	Glu	Chr03-17635594	3	17635594	1.43E-05	4.8
164	Glu	Chr03-57367207	3	57367207	2.61E-05	4.6
165	Glu	Chr04-9423787	4	9423787	2.17E-05	4.7
166	Glu	Chr04-10194634	4	10194634	4.3E-05	4.4
167	Glu	Chr04-10235767	4	10235767	2.5E-05	4.6
168	Glu	Chr04-25982717	4	25982717	8.68E-06	5.1
169	Glu	Chr04-100366722	4	100366722	7.49E-05	4.1
170	Glu	Chr05-143431993	5	143431993	3.07E-05	4.5
171	Glu	Chr05-159675841	5	159675841	7.33E-05	4.1
172	Glu	Chr05-166754628	5	166754628	1.49E-05	4.8
173	Glu	Chr06-81540072	6	81540072	5.9E-05	4.2
174	Glu	Chr06-280066668	6	280066668	5.84E-05	4.2
175	Glu	Chr07-70828704	7	70828704	4.75E-05	4.3
176	Glu	Chr07-158564034	7	158564034	4.33E-05	4.4
177	Gly	Chr01-48251669	1	48251669	2.12E-05	4.7
178	Gly	Chr01-92343722	1	92343722	7.17E-05	4.1
179	Gly	Chr02-32830249	2	32830249	6.74E-05	4.2
180	Gly	Chr02-260373089	2	260373089	7.17E-05	4.1
181	Gly	Chr02-260402443	2	260402443	1.36E-05	4.9
182	Gly	Chr02-260402474	2	260402474	7.11E-06	5.1
183	Gly	Chr02-260418229	2	260418229	3.55E-05	4.4

Sr No	Trait	SNP	Chr	Position	P.value	-log10
184	Gly	Chr02-260446649	2	260446649	2.86E-05	4.5
185	Gly	Chr03-195257204	3	195257204	7.59E-05	4.1
186	Gly	Chr03-197331468	3	197331468	3.31E-05	4.5
187	Gly	Chr04-24855828	4	24855828	6.02E-05	4.2
188	Gly	Chr04-156983332	4	156983332	7.18E-05	4.1
189	Gly	Chr04-163334205	4	163334205	5.97E-05	4.2
190	Gly	Chr05-163662767	5	163662767	1.28E-05	4.9
191	Gly	Chr06-141556368	6	141556368	1.68E-05	4.8
192	Gly	Chr06-147646969	6	147646969	7.37E-05	4.1
193	His	Chr01-46928608	1	46928608	4.36E-05	4.4
194	His	Chr01-89488582	1	89488582	2.15E-05	4.7
195	His	Chr01-93576262	1	93576262	6.4E-05	4.2
196	His	Chr01-94312547	1	94312547	5.5E-05	4.3
197	His	Chr01-226176848	1	226176848	1.29E-05	4.9
198	His	Chr02-260402443	2	260402443	2.28E-05	4.6
199	His	Chr03-212717051	3	212717051	2.97E-05	4.5
200	His	Chr07-2481826	7	2481826	4.22E-05	4.4
201	His	Chr07-67446316	7	67446316	2.57E-05	4.6
202	Ile	Chr02-30736113	2	30736113	5.85E-05	4.2
203	Ile	Chr05-147888048	5	147888048	6.94E-05	4.2
204	Ile	Chr06-44248544	6	44248544	3.01E-05	4.5
205	Leu	Chr03-63998406	3	63998406	7.13E-05	4.1
206	Leu	Chr03-322043558	3	322043558	6.82E-05	4.2
207	Leu	Chr05-140112743	5	140112743	4.43E-05	4.4
208	Leu	Chr05-147888048	5	147888048	5.23E-05	4.3
209	Leu	Chr06-44248544	6	44248544	1.03E-05	5.0
210	Leu	Chr07-274396150	7	274396150	5.77E-05	4.2
211	Lys	Chr01-63002928	1	63002928	3.56E-05	4.4
212	Lys	Chr01-90166787	1	90166787	2.34E-10	9.6
213	Lys	Chr01-91169824	1	91169824	7.27E-05	4.1
214	Lys	Chr01-91479838	1	91479838	7.27E-05	4.1
215	Lys	Chr01-94231924	1	94231924	1.76E-14	13.8
216	Lys	Chr02-121838429	2	121838429	5.97E-17	16.2
217	Lys	Chr02-239106627	2	239106627	6.36E-05	4.2
218	Lys	Chr02-239229714	2	239229714	2.42E-05	4.6
219	Lys	Chr03-60817768	3	60817768	7.45E-05	4.1
220	Lys	Chr03-138574287	3	138574287	4.74E-05	4.3
221	Lys	Chr04-45227929	4	45227929	4.71E-05	4.3
222	Lys	Chr04-77690978	4	77690978	6.32E-05	4.2
223	Lys	Chr04-77757362	4	77757362	5.78E-05	4.2
224	Lys	Chr05-93011085	5	93011085	5.08E-05	4.3
225	Lys	Chr05-164116688	5	164116688	4.68E-05	4.3
226	Lys	Chr06-109188248	6	109188248	5.6E-05	4.3
227	Lys	Chr06-172222184	6	172222184	1.22E-15	14.9
228	Lys	Chr06-205478857	6	205478857	4.98E-05	4.3
229	Lys	Chr06-205631671	6	205631671	2.41E-05	4.6

Sr No	Trait	SNP	Chr	Position	P.value	-log10
230	Lys	Chr06-235060792	6	235060792	3.02E-05	4.5
231	Lys	Chr07-84489	7	84489	2.89E-05	4.5
232	Lys	Chr07-196098235	7	196098235	1.91E-12	11.7
233	Lys	Chr07-224025869	7	224025869	5.13E-05	4.3
234	Met	Chr01-42575262	1	42575262	5.38E-05	4.3
235	Met	Chr01-49242723	1	49242723	1.03E-05	5.0
236	Met	Chr02-30079430	2	30079430	6.52E-05	4.2
237	Met	Chr02-43927562	2	43927562	2.55E-05	4.6
238	Met	Chr02-47134209	2	47134209	5.32E-05	4.3
239	Met	Chr02-70933243	2	70933243	9.75E-06	5.0
240	Met	Chr02-97664672	2	97664672	9.25E-06	5.0
241	Met	Chr02-103176518	2	103176518	6.73E-05	4.2
242	Met	Chr02-253088151	2	253088151	7.78E-05	4.1
243	Met	Chr02-267521396	2	267521396	1.01E-05	5.0
244	Met	Chr03-16509970	3	16509970	6.52E-05	4.2
245	Met	Chr03-16518726	3	16518726	1.78E-05	4.8
246	Met	Chr03-16532095	3	16532095	4.68E-05	4.3
247	Met	Chr03-36627361	3	36627361	2.46E-05	4.6
248	Met	Chr03-243699167	3	243699167	4.39E-06	5.4
249	Met	Chr03-316002106	3	316002106	5.15E-05	4.3
250	Met	Chr04-6908911	4	6908911	2.47E-07	6.6
251	Met	Chr04-12074362	4	12074362	4.45E-05	4.4
252	Met	Chr04-133790067	4	133790067	3.74E-05	4.4
253	Met	Chr04-140357459	4	140357459	1.31E-05	4.9
254	Met	Chr04-146132017	4	146132017	5.01E-05	4.3
255	Met	Chr04-150455099	4	150455099	6.87E-05	4.2
256	Met	Chr04-172145053	4	172145053	3.32E-05	4.5
257	Met	Chr05-22399993	5	22399993	1.95E-05	4.7
258	Met	Chr05-28823300	5	28823300	1.67E-07	6.8
259	Met	Chr05-54675887	5	54675887	6.08E-05	4.2
260	Met	Chr05-56381496	5	56381496	9.66E-06	5.0
261	Met	Chr05-109673968	5	109673968	4.77E-05	4.3
262	Met	Chr05-109881927	5	109881927	6.73E-05	4.2
263	Met	Chr05-144168157	5	144168157	3.17E-06	5.5
264	Met	Chr06-20133776	6	20133776	4.08E-05	4.4
265	Met	Chr06-58143188	6	58143188	4.89E-05	4.3
266	Met	Chr06-60135571	6	60135571	5.85E-05	4.2
267	Met	Chr06-139902830	6	139902830	6.73E-05	4.2
268	Met	Chr06-140184562	6	140184562	7.34E-05	4.1
269	Met	Chr06-178041770	6	178041770	4.52E-05	4.3
270	Met	Chr06-204881953	6	204881953	9.27E-06	5.0
271	Met	Chr06-244563760	6	244563760	2.89E-05	4.5
272	Met	Chr06-268068954	6	268068954	5.96E-05	4.2
273	Met	Chr07-33085807	7	33085807	5.43E-05	4.3
274	Met	Chr07-121902898	7	121902898	7.88E-05	4.1
275	Met	Chr07-227857258	7	227857258	4.64E-05	4.3

Sr No	Trait	SNP	Chr	Position	P.value	-log10
276	Met	Chr07-260308833	7	260308833	5.75E-05	4.2
277	Met	Chr07-261635550	7	261635550	3.22E-05	4.5
278	Met	Chr07-270998103	7	270998103	3.59E-06	5.4
279	Phe	Chr02-265919787	2	265919787	5.35E-05	4.3
280	Phe	Chr04-11760424	4	11760424	5.77E-05	4.2
281	Phe	Chr04-41164708	4	41164708	1.51E-05	4.8
282	Phe	Chr04-44260171	4	44260171	1.56E-05	4.8
283	Phe	Chr04-72939269	4	72939269	7.22E-05	4.1
284	Phe	Chr04-80514910	4	80514910	2.48E-05	4.6
285	Phe	Chr06-42701420	6	42701420	3.49E-05	4.5
286	Phe	Chr06-44248544	6	44248544	1.94E-05	4.7
287	Phe	Chr06-148161712	6	148161712	3.14E-05	4.5
288	Phe	Chr06-148593113	6	148593113	6.06E-05	4.2
289	Phe	Chr06-231042369	6	231042369	2.38E-05	4.6
290	Phe	Chr07-244969205	7	244969205	6.8E-05	4.2
291	Phe	Chr07-274396150	7	274396150	6.03E-05	4.2
292	Pro	Chr03-63998406	3	63998406	1.99E-05	4.7
293	Pro	Chr03-303065527	3	303065527	1.81E-05	4.7
294	Pro	Chr04-176129318	4	176129318	6.86E-05	4.2
295	Pro	Chr04-176130910	4	176130910	6.21E-05	4.2
296	Pro	Chr04-176235945	4	176235945	5.08E-05	4.3
297	Pro	Chr05-121930150	5	121930150	7.51E-05	4.1
298	Pro	Chr05-140112743	5	140112743	3.12E-06	5.5
299	Pro	Chr06-42701420	6	42701420	2.55E-06	5.6
300	Pro	Chr06-42720239	6	42720239	3.68E-05	4.4
301	Pro	Chr06-44248544	6	44248544	3.34E-06	5.5
302	Pro	Chr06-46345601	6	46345601	6.67E-05	4.2
303	Pro	Chr06-148593113	6	148593113	7.66E-05	4.1
304	Pro	Chr06-151157741	6	151157741	9.6E-06	5.0
305	Pro	Chr06-158395116	6	158395116	7.06E-05	4.2
306	Pro	Chr06-239715351	6	239715351	2E-05	4.7
307	Protein	Chr01-298443163	1	298443163	6.67E-05	4.2
308	Protein	Chr01-298452669	1	298452669	3.83E-05	4.4
309	Protein	Chr04-95901582	4	95901582	2.69E-05	4.6
310	Protein	Chr06-85758862	6	85758862	4.06E-05	4.4
311	Protein	Chr06-148801439	6	148801439	6.5E-06	5.2
312	Protein	Chr07-181103083	7	181103083	1.09E-05	5.0
313	Ser	Chr01-49129656	1	49129656	4.37E-05	4.4
314	Ser	Chr01-226869405	1	226869405	5.98E-05	4.2
315	Ser	Chr03-29975345	3	29975345	8.09E-06	5.1
316	Ser	Chr03-70834875	3	70834875	5.71E-05	4.2
317	Ser	Chr03-123369281	3	123369281	1.5E-05	4.8
318	Ser	Chr03-125124951	3	125124951	2.5E-05	4.6
319	Ser	Chr03-131572236	3	131572236	3.88E-05	4.4
320	Ser	Chr03-138855567	3	138855567	1.16E-05	4.9
321	Ser	Chr03-152794146	3	152794146	2.37E-05	4.6

Sr No	Trait	SNP	Chr	Position	P.value	-log10
322	Ser	Chr03-159973304	3	159973304	2.74E-05	4.6
323	Ser	Chr03-178267540	3	178267540	7.31E-05	4.1
324	Ser	Chr04-10235767	4	10235767	1.89E-05	4.7
325	Ser	Chr04-24816491	4	24816491	3.86E-05	4.4
326	Ser	Chr04-24889383	4	24889383	7.22E-05	4.1
327	Ser	Chr04-24900886	4	24900886	2.45E-06	5.6
328	Ser	Chr04-236999746	4	236999746	8.29E-06	5.1
329	Ser	Chr04-238806281	4	238806281	1.86E-05	4.7
330	Ser	Chr05-106999828	5	106999828	5.67E-06	5.2
331	Ser	Chr06-3098168	6	3098168	3.92E-05	4.4
332	Ser	Chr06-40295330	6	40295330	5.3E-05	4.3
333	Ser	Chr06-42671475	6	42671475	1.56E-05	4.8
334	Ser	Chr06-85240212	6	85240212	6E-05	4.2
335	Ser	Chr06-141278568	6	141278568	3.07E-06	5.5
336	Ser	Chr06-141365243	6	141365243	5.36E-05	4.3
337	Ser	Chr06-141369306	6	141369306	6.8E-05	4.2
338	Ser	Chr06-142083538	6	142083538	6.47E-05	4.2
339	Ser	Chr06-142083545	6	142083545	5.07E-05	4.3
340	Ser	Chr06-142217225	6	142217225	2.07E-05	4.7
341	Ser	Chr06-142296880	6	142296880	4.17E-06	5.4
342	Ser	Chr06-142355538	6	142355538	3.53E-05	4.5
343	Ser	Chr06-143916622	6	143916622	1.27E-05	4.9
344	Ser	Chr06-143959208	6	143959208	3.26E-05	4.5
345	Ser	Chr06-144042991	6	144042991	3.67E-05	4.4
346	Ser	Chr06-144184599	6	144184599	2.03E-05	4.7
347	Ser	Chr06-144247852	6	144247852	4.5E-05	4.3
348	Ser	Chr06-145381624	6	145381624	3.74E-05	4.4
349	Ser	Chr06-147238506	6	147238506	6.82E-05	4.2
350	Ser	Chr06-147646969	6	147646969	9.82E-07	6.0
351	Ser	Chr06-147791094	6	147791094	7.66E-05	4.1
352	Ser	Chr06-148448308	6	148448308	6.7E-05	4.2
353	Ser	Chr06-148456126	6	148456126	5.23E-05	4.3
354	Ser	Chr06-148472676	6	148472676	7.14E-05	4.1
355	Ser	Chr06-148494005	6	148494005	3.78E-05	4.4
356	Ser	Chr06-148728038	6	148728038	7.33E-05	4.1
357	Ser	Chr06-148811602	6	148811602	4.1E-05	4.4
358	Ser	Chr06-149010507	6	149010507	3.53E-05	4.5
359	Ser	Chr06-149700499	6	149700499	4.56E-06	5.3
360	Ser	Chr06-149700872	6	149700872	6.92E-05	4.2
361	Ser	Chr06-150029374	6	150029374	6.7E-05	4.2
362	Ser	Chr06-150403027	6	150403027	2.97E-05	4.5
363	Ser	Chr06-150474737	6	150474737	7.9E-05	4.1
364	Ser	Chr06-150481947	6	150481947	3.31E-05	4.5
365	Ser	Chr06-150485520	6	150485520	6.11E-05	4.2
366	Ser	Chr06-150494777	6	150494777	6.86E-05	4.2
367	Ser	Chr06-150525488	6	150525488	5.11E-06	5.3

Sr No	Trait	SNP	Chr	Position	P.value	-log10
368	Ser	Chr06-150525524	6	150525524	3.75E-05	4.4
369	Ser	Chr06-150978580	6	150978580	3.45E-05	4.5
370	Ser	Chr06-150993846	6	150993846	1.4E-05	4.9
371	Ser	Chr06-151675083	6	151675083	7.04E-06	5.2
372	Ser	Chr06-151706762	6	151706762	7.3E-05	4.1
373	Ser	Chr06-151719358	6	151719358	8.23E-06	5.1
374	Ser	Chr06-151719367	6	151719367	1.71E-05	4.8
375	Ser	Chr06-152682693	6	152682693	3.13E-05	4.5
376	Ser	Chr06-152799023	6	152799023	5.03E-05	4.3
377	Ser	Chr06-152851965	6	152851965	7.33E-05	4.1
378	Ser	Chr06-152871312	6	152871312	1.09E-05	5.0
379	Ser	Chr06-153139197	6	153139197	7.29E-05	4.1
380	Ser	Chr06-153773882	6	153773882	2.6E-05	4.6
381	Ser	Chr06-153800333	6	153800333	1.55E-05	4.8
382	Ser	Chr06-153801124	6	153801124	4.73E-05	4.3
383	Ser	Chr06-153943117	6	153943117	6.97E-05	4.2
384	Ser	Chr06-154131992	6	154131992	4.71E-07	6.3
385	Ser	Chr06-154143999	6	154143999	1.23E-05	4.9
386	Ser	Chr06-154180998	6	154180998	5.88E-05	4.2
387	Ser	Chr06-154263091	6	154263091	1.08E-05	5.0
388	Ser	Chr06-154287095	6	154287095	7.1E-06	5.1
389	Ser	Chr06-154333801	6	154333801	2.79E-06	5.6
390	Ser	Chr06-154333847	6	154333847	2.3E-05	4.6
391	Ser	Chr06-154418575	6	154418575	2.46E-05	4.6
392	Ser	Chr06-154438582	6	154438582	1.69E-05	4.8
393	Ser	Chr06-154482007	6	154482007	5.08E-05	4.3
394	Ser	Chr06-154869029	6	154869029	3.2E-05	4.5
395	Ser	Chr06-155109345	6	155109345	2.36E-05	4.6
396	Ser	Chr06-155111082	6	155111082	7.8E-05	4.1
397	Ser	Chr06-155130749	6	155130749	4.67E-05	4.3
398	Ser	Chr06-155143276	6	155143276	1.2E-05	4.9
399	Ser	Chr06-155521958	6	155521958	4.32E-05	4.4
400	Ser	Chr06-155521965	6	155521965	1.68E-05	4.8
401	Ser	Chr06-155600577	6	155600577	2.4E-05	4.6
402	Ser	Chr06-155600625	6	155600625	1.04E-05	5.0
403	Ser	Chr06-155605418	6	155605418	5.59E-06	5.3
404	Ser	Chr06-155762384	6	155762384	4.58E-05	4.3
405	Ser	Chr06-155865607	6	155865607	4.95E-05	4.3
406	Ser	Chr06-155914941	6	155914941	4.81E-05	4.3
407	Ser	Chr06-155914948	6	155914948	5.36E-05	4.3
408	Ser	Chr06-155914982	6	155914982	2.72E-05	4.6
409	Ser	Chr06-155959930	6	155959930	6.62E-05	4.2
410	Ser	Chr06-155969741	6	155969741	3.1E-05	4.5
411	Ser	Chr06-155975002	6	155975002	2.71E-05	4.6
412	Ser	Chr06-156019162	6	156019162	5.11E-05	4.3
413	Ser	Chr06-156088989	6	156088989	2.13E-05	4.7

Sr No	Trait	SNP	Chr	Position	P.value	-log10
414	Ser	Chr06-156205574	6	156205574	1.39E-05	4.9
415	Ser	Chr06-159761763	6	159761763	9.75E-06	5.0
416	Ser	Chr06-170860403	6	170860403	5.53E-05	4.3
417	Ser	Chr06-181901879	6	181901879	5.04E-05	4.3
418	Ser	Chr06-273564950	6	273564950	6.47E-05	4.2
419	Thr	Chr02-64937555	2	64937555	5.83E-05	4.2
420	Thr	Chr03-72933205	3	72933205	4.8E-05	4.3
421	Thr	Chr03-300002014	3	300002014	7.4E-05	4.1
422	Thr	Chr03-324858012	3	324858012	7.75E-05	4.1
423	Thr	Chr04-39197650	4	39197650	3.57E-05	4.4
424	Thr	Chr04-44286610	4	44286610	5.38E-05	4.3
425	Thr	Chr04-176129247	4	176129247	4.8E-05	4.3
426	Thr	Chr04-176129318	4	176129318	7.31E-05	4.1
427	Thr	Chr04-176457678	4	176457678	3.15E-05	4.5
428	Thr	Chr04-222400380	4	222400380	6.31E-05	4.2
429	Thr	Chr04-239821629	4	239821629	2.39E-05	4.6
430	Thr	Chr05-14919841	5	14919841	1.02E-05	5.0
431	Thr	Chr05-56381500	5	56381500	2.76E-05	4.6
432	Thr	Chr05-124307775	5	124307775	3.58E-05	4.4
433	Thr	Chr06-42671475	6	42671475	1.36E-05	4.9
434	Thr	Chr06-42701420	6	42701420	6.99E-05	4.2
435	Thr	Chr06-42720239	6	42720239	2.59E-05	4.6
436	Thr	Chr06-43329491	6	43329491	5.49E-05	4.3
437	Thr	Chr06-48239400	6	48239400	3.28E-05	4.5
438	Thr	Chr06-59405668	6	59405668	7.62E-05	4.1
439	Thr	Chr06-141278568	6	141278568	3.88E-05	4.4
440	Thr	Chr06-142217225	6	142217225	5.38E-05	4.3
441	Thr	Chr06-142296880	6	142296880	2.43E-05	4.6
442	Thr	Chr06-144093518	6	144093518	2.59E-05	4.6
443	Thr	Chr06-147646969	6	147646969	3.88E-05	4.4
444	Thr	Chr06-148161712	6	148161712	3.96E-05	4.4
445	Thr	Chr06-148593113	6	148593113	7.55E-05	4.1
446	Thr	Chr06-151456453	6	151456453	5.27E-05	4.3
447	Thr	Chr06-151719358	6	151719358	6.53E-05	4.2
448	Thr	Chr06-154131992	6	154131992	3.55E-06	5.4
449	Thr	Chr06-154438582	6	154438582	6.1E-05	4.2
450	Thr	Chr06-155914982	6	155914982	2.49E-05	4.6
451	Thr	Chr06-156019162	6	156019162	5.02E-05	4.3
452	Thr	Chr06-156072519	6	156072519	5.3E-05	4.3
453	Trp	Chr01-6790832	1	6790832	4.37E-05	4.4
454	Trp	Chr01-10125250	1	10125250	6.85E-05	4.2
455	Trp	Chr01-185333999	1	185333999	4.04E-05	4.4
456	Trp	Chr01-185337296	1	185337296	3.98E-05	4.4
457	Trp	Chr01-185346780	1	185346780	2.89E-05	4.5
458	Trp	Chr01-185349009	1	185349009	2.48E-05	4.6
459	Trp	Chr01-185349024	1	185349024	1.33E-05	4.9

Sr No	Trait	SNP	Chr	Position	P.value	-log10
460	Trp	Chr01-185349336	1	185349336	6.12E-05	4.2
461	Trp	Chr01-185352338	1	185352338	4.1E-05	4.4
462	Trp	Chr01-185366740	1	185366740	4.17E-05	4.4
463	Trp	Chr01-185367103	1	185367103	1.48E-05	4.8
464	Trp	Chr01-185367122	1	185367122	5.38E-05	4.3
465	Trp	Chr01-185374632	1	185374632	6.08E-05	4.2
466	Trp	Chr01-185388015	1	185388015	1.75E-05	4.8
467	Trp	Chr01-185388040	1	185388040	6.63E-05	4.2
468	Trp	Chr01-185388216	1	185388216	5.85E-05	4.2
469	Trp	Chr01-185388363	1	185388363	6.72E-05	4.2
470	Trp	Chr01-185389090	1	185389090	2.07E-05	4.7
471	Trp	Chr01-185467654	1	185467654	3.74E-05	4.4
472	Trp	Chr01-185467706	1	185467706	6.72E-05	4.2
473	Trp	Chr01-185470276	1	185470276	4.14E-06	5.4
474	Trp	Chr01-230866409	1	230866409	1.14E-05	4.9
475	Trp	Chr01-262089511	1	262089511	5.22E-05	4.3
476	Trp	Chr01-272133009	1	272133009	7.51E-05	4.1
477	Trp	Chr02-42992926	2	42992926	3.68E-05	4.4
478	Trp	Chr02-49085401	2	49085401	7.25E-05	4.1
479	Trp	Chr02-107664963	2	107664963	5.92E-05	4.2
480	Trp	Chr02-195405434	2	195405434	6.96E-05	4.2
481	Trp	Chr02-249286963	2	249286963	6.79E-05	4.2
482	Trp	Chr02-260977098	2	260977098	1.39E-05	4.9
483	Trp	Chr03-72850957	3	72850957	2.12E-09	8.7
484	Trp	Chr03-271904284	3	271904284	6.6E-05	4.2
485	Trp	Chr04-67844255	4	67844255	6.11E-05	4.2
486	Trp	Chr04-139361713	4	139361713	1.6E-05	4.8
487	Trp	Chr04-172469099	4	172469099	2.16E-05	4.7
488	Trp	Chr04-229687210	4	229687210	6.7E-05	4.2
489	Trp	Chr05-56191227	5	56191227	4.47E-08	7.3
490	Trp	Chr05-85335264	5	85335264	1.2E-05	4.9
491	Trp	Chr05-85365470	5	85365470	2.77E-05	4.6
492	Trp	Chr05-85365521	5	85365521	3.16E-05	4.5
493	Trp	Chr05-85385249	5	85385249	3E-05	4.5
494	Trp	Chr05-85385265	5	85385265	3E-05	4.5
495	Trp	Chr05-85390559	5	85390559	7.3E-06	5.1
496	Trp	Chr05-130399973	5	130399973	5.98E-05	4.2
497	Trp	Chr05-130400756	5	130400756	6.79E-05	4.2
498	Trp	Chr05-130405914	5	130405914	5.06E-05	4.3
499	Trp	Chr05-130407209	5	130407209	6.79E-05	4.2
500	Trp	Chr05-156277752	5	156277752	7.67E-05	4.1
501	Trp	Chr06-16884709	6	16884709	4.36E-05	4.4
502	Trp	Chr06-49565280	6	49565280	2.77E-05	4.6
503	Trp	Chr06-249278794	6	249278794	1.96E-13	12.7
504	Trp	Chr07-27440757	7	27440757	1.77E-05	4.8
505	Trp	Chr07-33954050	7	33954050	3.88E-05	4.4

Sr No	Trait	SNP	Chr	Position	P.value	-log10
506	Trp	Chr07-52388267	7	52388267	6.95E-05	4.2
507	Trp	Chr07-52818587	7	52818587	3.24E-05	4.5
508	Tyr	Chr01-92248603	1	92248603	7.73E-05	4.1
509	Tyr	Chr01-181871736	1	181871736	2.94E-05	4.5
510	Tyr	Chr02-38379572	2	38379572	3.67E-05	4.4
511	Tyr	Chr02-38447267	2	38447267	4.05E-05	4.4
512	Tyr	Chr02-38450897	2	38450897	6.38E-05	4.2
513	Tyr	Chr02-38451180	2	38451180	6.38E-05	4.2
514	Tyr	Chr02-66144770	2	66144770	7.22E-05	4.1
515	Tyr	Chr02-260373089	2	260373089	5.17E-05	4.3
516	Tyr	Chr02-265919787	2	265919787	5.96E-05	4.2
517	Tyr	Chr03-212717051	3	212717051	6.66E-05	4.2
518	Tyr	Chr04-31234651	4	31234651	8.16E-06	5.1
519	Tyr	Chr04-41164708	4	41164708	4.51E-05	4.3
520	Tyr	Chr04-48751716	4	48751716	5.5E-05	4.3
521	Tyr	Chr04-63781152	4	63781152	6.47E-06	5.2
522	Tyr	Chr04-80514910	4	80514910	4.2E-05	4.4
523	Tyr	Chr05-34555542	5	34555542	4.8E-06	5.3
524	Tyr	Chr05-66984328	5	66984328	6.66E-05	4.2
525	Tyr	Chr06-42701420	6	42701420	6.22E-05	4.2
526	Tyr	Chr06-72189581	6	72189581	3.69E-05	4.4
527	Tyr	Chr06-170400800	6	170400800	6.88E-05	4.2
528	Tyr	Chr06-175526772	6	175526772	5.52E-05	4.3
529	Tyr	Chr07-11929084	7	11929084	7.86E-05	4.1
530	Tyr	Chr07-11929673	7	11929673	7.86E-05	4.1
531	Tyr	Chr07-11953597	7	11953597	7.86E-05	4.1
532	Tyr	Chr07-142336899	7	142336899	4.92E-05	4.3
533	Tyr	Chr07-197164446	7	197164446	4.64E-05	4.3
534	Tyr	Chr07-199205536	7	199205536	3.74E-05	4.4
535	Val	Chr01-215572339	1	215572339	6.14E-05	4.2
536	Val	Chr01-215577764	1	215577764	4.04E-05	4.4
537	Val	Chr02-30736113	2	30736113	7.49E-05	4.1
538	Val	Chr02-130108217	2	130108217	4.24E-05	4.4
539	Val	Chr02-260587678	2	260587678	1.75E-05	4.8
540	Val	Chr03-322043558	3	322043558	1.06E-05	5.0
541	Val	Chr04-11760424	4	11760424	7.05E-05	4.2
542	Val	Chr05-77862672	5	77862672	5.74E-05	4.2
543	Val	Chr06-44248544	6	44248544	1.69E-05	4.8
544	Val	Chr07-274396150	7	274396150	4.13E-05	4.4

Histidine (His), Isoleucine (Ile), Leucine (Leu), Lysine (Lys), Methionine (Met), Phenylalanine (Phe), Threonine (Thr), Tryptophan (Trp), Valine (Val), Alanine (Ala), Arginine (Arg), Asparatate (Asp), Cysteine (Cys), Glutamic Acid (Glu), Glycine (Gly), Proline (Pro), Serine (Ser), Tyrosine (Tyr)
