# Supplementary material for: Genome-wide SNPs and candidate genes underlying the genetic variations for protein and amino acids in pearl millet (Pennisetum glaucum) germplasm
Source: Planta. 2024 Jul 27;260(3):63. doi: 10.1007/s00425-024-04495-y (PMC11283402; doi:10.1007/s00425-024-04495-y)
Supplement: Supplementary file 7 — Supplementary file7 (PDF 726 KB) [file 425_2024_4495_MOESM7_ESM.pdf]

**Genome-wide SNPs and candidate genes underlying the genetic variations for protein and amino acids in pearl millet (*Pennisetum glaucum*) germplasm**

**PLANTA**


**Satbeer Singh[1,2], Chandra Bhan Yadav[1,3], Nelson Lubanga[1], Matthew Hegarty[1], Rattan S. Yadav[1]***

[1] Institute of Biological Environmental and Rural Sciences (IBERS), Aberystwyth University, Aberystwyth, SY23 3EE, United Kingdom

[2] Division of Agrotechnology, Council of Scientific and Industrial Research (CSIR) - Institute of Himalayan Bioresource Technology, Palampur, Himachal Pradesh 176 061, India

[3] Department of Genetics, Genomics, and Breeding, NIAB-EMR, East Malling, ME19 6BJ, United Kingdom

* Corresponding author: rsy@aber.ac.uk


**Online Resource S7** Q-Q plots of marker trait association for all traits

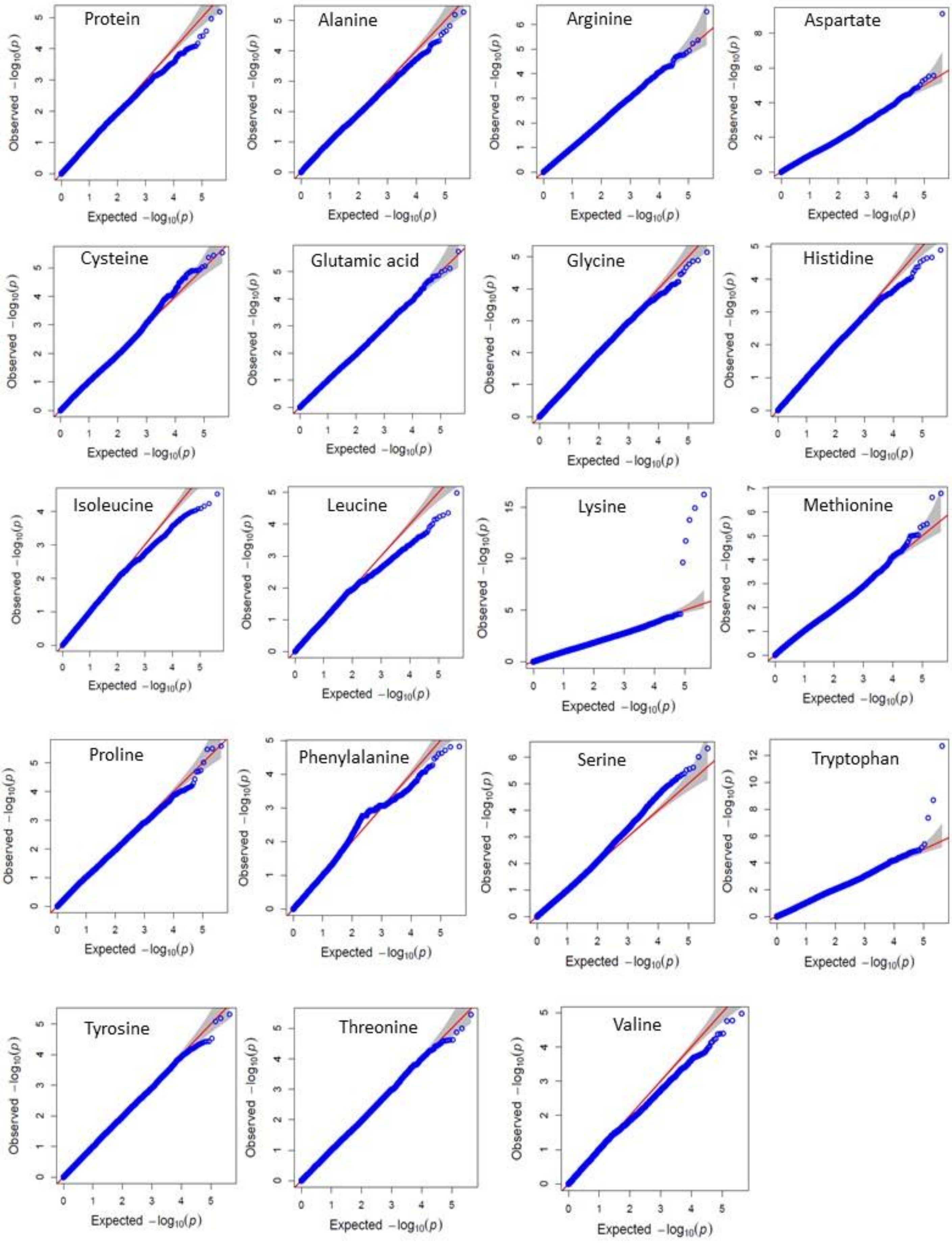
Protein
Alanine
Arginine
Aspartate
Cysteine
Glutamic acid
Glycine
Histidine
Isoleucine
Leucine
Lysine
Methionine
Proline
Phenylalanine
Serine
Tryptophan
Tyrosine
Threonine
Valine
Observed $-\log_{10}(p)$
Expected $-\log_{10}(p)$
