# Supplementary material for: Genome-wide SNPs and candidate genes underlying the genetic variations for protein and amino acids in pearl millet (Pennisetum glaucum) germplasm
Source: Planta. 2024 Jul 27;260(3):63. doi: 10.1007/s00425-024-04495-y (PMC11283402; doi:10.1007/s00425-024-04495-y)
Supplement: Supplementary file 8 — Supplementary file8 (PDF 487 KB) [file 425_2024_4495_MOESM8_ESM.pdf]

Genome-wide SNPs and candidate genes underlying the genetic variations for protein and amino acids in pearl millet (*Pennisetum glaucum*) germplasm

PLANTA

Satbeer Singh[1,2], Chandra Bhan Yadav[1,3], Nelson Lubanga[1], Matthew Hegarty[1], Rattan S. Yadav[1]*

[1] Institute of Biological Environmental and Rural Sciences (IBERS), Aberystwyth University, Aberystwyth, SY23 3EE, United Kingdom

[2] Division of Agrotechnology, Council of Scientific and Industrial Research (CSIR) - Institute of Himalayan Bioresource Technology, Palampur, Himachal Pradesh 176 061, India

[3] Department of Genetics, Genomics, and Breeding, NIAB-EMR, East Malling, ME19 6BJ, United Kingdom

* Corresponding author: rsy@aber.ac.uk

Online Resource S8 Candidate genes near significant SNPs

Sr No	Chr	SNP Position	Gene ID	Gene description
1	1	6790832	dpca1g004440.840	Nodescriptionavailable(gfam005176)|ProteincontainingKelch_1,Kelch_2,Kelch_3,Kelch_4,Kelch_5,Kelch_6,PCRF,RF-1domains
2	1	10125250	dpca1g005780.840	receptor-likeprotein12-like(gfam020660)|ProteincontainingLRRNT_2,LRR_1,LRR_4,LRR_6,LRR_8domains
3	1	42575262	dpca1g015490.840	Lecithin-cholesterolacyltransferase-like1
4	1	46928608	dpca1g016880.840	UPF0481 protein At3g47200-like isoformX2 (gfam000134)|Protein containing DUF247 domains
5	1	49129656	dpca1g017700.840	cytochrome P45071A1-like, partial (gfam011650)|Protein containing p450 domains
6	1	49242723	dpca1g017760.840	Protein containing DUF724 domains
7	1	55371493	dpca1g019660.840	Uncharacterized protein (Fragment)
8	1	63002928	dpca1g021980.840	probable inactivepatatin-likeprotein9 (gfam000336)|Protein containing Patatin domains
9	1	64427363	dpca1g022450.840	Alpha-amylase-like2isoform1(gfam001493)|ProteincontainingAlpha-amylase,Alpha-amyl_C2domains
10	1	64687910	dpca1g022460.840	BRI1kinaseinhibitor1
11	1	89488582	dpca1g030000.840	Nodescriptionavailable(gfam004615)|ProteincontainingPPR,PPR_1,PPR_2,PPR_3,PPR_long,RPM2,TPR_14,TPR_19,TPR_7domains
12	1	90167351	dpca1g030220.840	KIAA1076protein(Fragment)(gfam067258)|ProteincontainingPre-SET,SAD_SRA,SETdomains
13	1	90203370	dpca1g030260.840	cleavage and polyadenylation specificity factor subunit5-likeisoformX2 (gfam002613) ProteincontainingNUDIX_2domains
14	1	91169824	dpca1g030490.840	protein MBTB40-Bric-a-Brac,Tramtrack, Broad Complex BTB domain with Meprin and TRAF Homology MATH domain (gfam000127)|ProteincontainingBTBdomains
15	1	91479838	dpca1g030540.840	ProteincontainingDUF755domains
16	1	91831977	dpca1g030620.840	Descriptionnotavailable
17	1	92027967	dpca1g030640.840	proteinTHION1-Plantthioninfamilyproteinprecursor,putative(gfam004711)|ProteincontainingThionindomains
18	1	92248603	dpca1g030710.840	proteinTHION1-Plantthioninfamilyproteinprecursor,putative(gfam004711)|ProteincontainingThionindomains
19	1	92343722	dpca1g030760.840	proteinTHION1-Plantthioninfamilyproteinprecursor,putative(gfam004711)|ProteincontainingThionindomains
20	1	93576262	dpca1g031030.840	progastricsin(pepsinogenC)(gfam000023)|ProteincontainingAsp,TAXi_C,TAXi_Ndomains
21	1	93641283	dpca1g031050.840	gamma-terpinenesynthase,chloroplastic-likeisoformX3(gfam000204)|ProteincontainingTerpene_synth,Terpene_synth_Cdomains
22	1	93900332	dpca1g031080.840	Os05g0517400protein
23	1	94231924	dpca1g031190.840	ProteincontainingABC_tran_CTD,Snapin_Pallidin,Syntaxin_2domains

Sr No	Chr	SNP Position	Gene ID	Gene description
24	1	94312547	dpca1g031210.840	cytochromeP4503A30-like(gfam005628)|Proteincontainingp450domains
25	1	107682403	dpca1g033880.840	FunctionunknownXXXXX(gfam010611)
26	1	119027870	dpca1g035540.840	Retrotransposonprotein,putative,unclassified
27	1	130249468	dpca1g037040.840	Descriptionnotavailable
28	1	140766648	dpca1g038630.840	lipoxygenase6,chloroplastic(gfam000335)|Protein containingLipoxygenase,PLATdomains
29	1	175920885	dpca1g044570.840	ProteincontainingDUF3511domains
30	1	181871736	dpca1g046390.840	Nodescriptionavailable(gfam032847)|ProteincontainingHexokinase_1,Hexokinase_2domains
31	1	185139326	dpca1g047560.840	cyclin-dependentkinaseC-1isoformX1
32	1	185349024	dpca1g047610.840	PREDICTED:phospholipaseA(1)DAD1,chloroplastic(gfam000448)|ProteincontainingLipase_3domains
33	1	185352338	dpca1g047630.840	Uncharacterizedprotein
34	1	185470276	dpca1g047650.840	ProteincontainingDUF588domains
35	1	195681683	dpca1g049880.840	Milt1aputativepolyprotein
36	1	215572339	dpca1g055320.840	Uncharacterizedprotein
37	1	215577764	dpca1g055330.840	Uncharacterizedprotein
38	1	217678759	dpca1g055980.840	Exocystsubunitexo70familyproteinA1(gfam000222)|ProteincontainingExo70domains
39	1	226176848	dpca1g058460.840	Uncharacterizedprotein
40	1	226869405	dpca1g058590.840	FunctionunknownXXXXX(gfam010611)
41	1	228026675	dpca1g058860.840	ProteincontainingMethyltransf_11domains
42	1	230866409	dpca1g059800.840	ProteincontainingN2227domains
43	1	241192934	dpca1g062110.840	Indole-3-acetatebeta-glucosyltransferaseXnoC3(gfam009042)
44	1	261635663	dpca1g068790.840	Nucleosidediphosphatekinase(gfam035291)|ProteincontainingNDKdomains
45	1	262089511	dpca1g069130.840	Zincfingerfamilyproteinisoform1(gfam003530)|ProteincontainingPHD,Prok-RING_1,Prok-RING_4,SAD_SRA,zf-C3HC4,zf-C3HC4_2,zf-C3HC4_3,zf-C3HC4_4,zf-RING_2,zf-RING_5,zf-RING_UBOXdomains
46	1	272133009	dpca1g072970.840	BifunctionalproteinFolD(gfam001146)|ProteincontainingTHF_DHG_CYH,THF_DHG_CYH_Cdomains
47	1	272773528	dpca1g073290.840	proteinOsDegp10-PutativeDegproteasehomologue,expressed(gfam001228)|ProteincontainingPDZ_2,PDZ_3,PDZ_6,Trypsin,Trypsin_2domains
48	1	272828045	dpca1g073290.840	proteinOsDegp10-PutativeDegproteasehomologue,expressed(gfam001228)|ProteincontainingPDZ_2,PDZ_3,PDZ_6,Trypsin,Trypsin_2domains
49	1	277783844	dpca1g075320.840	galactosylgalactosylxylosylprotein3-beta-glucuronosyltransferase2(gfam000668)|ProteincontainingGlyco_transf_43domains
50	1	283097941	dpca1g077850.840	proteinexpressedprotein(gfam033269)|ProteincontainingFH2domains
51	1	285641243	dpca1g078980.840	Transcriptionfactor,putative5NpXX(gfam000270)

Sr No	Chr	SNP Position	Gene ID	Gene description
52	1	298411919	dpca1g086920.840	ProteincontainingRibosomal_L10,RL10P_insertdomains
53	1	298443163	dpca1g086960.840	PREDICTED:ubiquitin-likemodifier-activatingenzyme7isoformX2(gfam001074)|ProteincontainingE1_4HB,E1_FCCH,E1_UFD,ThiF,UBA_e1_thiolCysdomains
54	1	298452669	dpca1g086970.840	Trichomebirefringence-like33isoform1(gfam000089)|ProteincontainingPC-Esterase,PMR5Ndomains
55	2	2669720	dpca2g089740.840	Os05g0127200protein
56	2	20214476	dpca2g099410.840	ULP_PROTEASEdomain-containingprotein
57	2	30079430	dpca2g103830.840	PREDICTED:proteinFAR1-RELATEDSEQUENCE4isoformX3(gfam000167)|ProteincontainingFAR1,MULE,SWIMdomains
58	2	30736113	dpca2g104080.840	ProteincontainingInhibitor_I29domains
59	2	32830249	dpca2g104920.840	Nodescriptionavailable(gfam004969)|Proteincontainingzf-C3Hc3Hdomains
60	2	37483579	dpca2g106650.840	Functionunknown5NOC3(gfam001815)
61	2	38379572	dpca2g106900.840	Uncharacterizedprotein
62	2	38447267	dpca2g106940.840	RNP-1likeRNA-bindingprotein(gfam080853)|ProteincontainingRRM_1domains
63	2	38451180	dpca2g106930.840	ProteincontainingNeprosindomains
64	2	39671080	dpca2g107500.840	PREDICTED:generaltranscriptionfactor3Cpolypeptide5-likeisoformX1(gfam008217)|ProteincontainingTau95,Tau95_Ndomains
65	2	42992926	dpca2g108660.840	Uncharacterizedprotein
66	2	43578448	dpca2g108860.840	Nodescriptionavailable(gfam009048)|ProteincontainingDUF3368,PPR,PPR_1,PPR_2,PPR_3,PPR_long,TPR_10,TPR_14,TPR_19,TPR_2,TPR_7domains
67	2	43927562	dpca2g109130.840	ProteincontainingCSDdomains
68	2	47134209	dpca2g110500.840	Cytokininoxidase2
69	2	49085401	dpca2g111230.840	Uncharacterizedprotein(gfam000059)|Proteincontainingadh_short,adh_short_C2,KR,NAD_binding_10domains
70	2	64937555	dpca2g117570.840	NADdependentepimerase/dehydratasefamilyprotein(gfam000045)|Proteincontaining3Beta_HSD,Epimerase,GDP_Man_Dehyd,NAD_binding_10,RmlD_sub_binddomains
71	2	66144770	dpca2g118060.840	Inorganicpyrophosphatase(gfam034382)|ProteincontainingPyrophosphatase,zf-RING_2,zf-RING_UBOXdomains
72	2	70933243	dpca2g119890.840	Uncharacterizedprotein(Fragment)
73	2	93450528	dpca2g128300.840	RibosomalproteinS30(gfam004440)|ProteincontainingRibosomal_S30domains
74	2	97664672	dpca2g129980.840	IsopenicillinNsynthase(gfam000011)|Proteincontaining2OG-FeII_Oxy,DIOX_Ndomains
75	2	103176518	dpca2g131840.840	Putativepolyprotein
76	2	107664963	dpca2g132980.840	Functionunknown5NOC3(gfam003005)|ProteincontainingPAN_4domains

Sr No	Chr	SNP Position	Gene ID	Gene description
77	2	118163944	dpca2g135970.840	FunctionunknownXXXXX(gfam010611)
78	2	118410674	dpca2g136090.840	Aldehydedehydrogenase2-3
79	2	121838429	dpca2g137280.840	ProteinthatbindstoFpr1p(FKBP12),conferringrapamycinresistancebycompetingwithrapamycinforFpr1pbinding,hassimilaritytoputativetranscriptionfactors,includingD.melanogastershuttlecraftandhumanNFX1(gfam005585)|ProteincontainingR3H,zf-NF-X1domains
80	2	130108217	dpca2g139180.840	Nodescriptionavailable(gfam009048)|ProteincontainingPPR,PPR_1,PPR_2,PPR_3domains
81	2	156304901	dpca2g143560.840	TranscriptionfactorMYB425NOC3(gfam000008)|ProteincontainingMyb_DNA-binding,Myb_DNA-bind_6domains
82	2	193359922	dpca2g149380.840	ProteincontainingF-box,F-box-likedomains
83	2	195405434	dpca2g149690.840	bacterial-likefructose-1,6-bisphosphataseXXXXX(gfam001743)|ProteincontainingTHOC7domains
84	2	195974730	dpca2g149790.840	Nodescriptionavailable(gfam009485)|ProteincontainingPPR,PPR_2domains
85	2	196670206	dpca2g149910.840	PREDICTED:plasmamembrane-associatedcation-bindingprotein1-likeisoform2(gfam005239)|ProteincontainingDREPPdomains
86	2	209374964	dpca2g152960.840	PREDICTED:probableproteinphosphatase2C8(gfam000027)|ProteincontainingPP2C,PP2C_2domains
87	2	228572807	dpca2g157970.840	Functionunknown5NOC3(gfam001452)
88	2	239229714	dpca2g160990.840	proteinYIF1Bisoform7(gfam002023)|ProteincontainingYIF1,Yip1domains
89	2	239573425	dpca2g161180.840	60SribosomalproteinL36(gfam001447)|ProteincontainingRibosomal_L36edomains
90	2	249286963	dpca2g164190.840	Nodescriptionavailable(gfam017829)|ProteincontainingGamma-thionin,SLR1-BPdomains
91	2	253088151	dpca2g165330.840	ProteincontainingBeach,DUF4704,Laminin_G_3,Pentaxin,PH_BEACHdomains
92	2	253101995	dpca2g165340.840	Uncharacterizedprotein
93	2	258586012	dpca2g168280.840	proteinOsFBX390-F-boxdomaincontainingprotein(gfam000010)|ProteincontainingF-box,F-box-likedomains
94	2	260373089	dpca2g169290.840	Elongationfactor1-alpha(Fragment)(gfam000244)|ProteincontainingGTP_EFTU,GTP_EFTU_D2,GTP_EFTU_D3,GTP_EFTU_D4,MMR_HSR1domains
95	2	260402443	dpca2g169300.840	Elongationfactor1-alpha(Fragment)(gfam000244)|ProteincontainingGTP_EFTU,GTP_EFTU_D2,GTP_EFTU_D3,GTP_EFTU_D4,MMR_HSR1domains
96	2	260587678	dpca2g169390.840	PREDICTED:probablereceptor-likeproteinkinaseAt1g67000(gfam009501)|ProteincontainingLRRNT_2,LRR_1,LRR_4,LRR_6,LRR_8,Pkinase,Pkinase_Tyrdomains
97	2	260977098	dpca2g169490.840	PeptidaseC1A,papain(gfam008941)|ProteincontainingPeptidase_C1domains

Sr No	Chr	SNP Position	Gene ID	Gene description
98	2	265087146	dpca2g170830.840	Os03g0156800protein
99	2	265919787	dpca2g171080.840	Functionunknown5NOC3(gfam020819)|ProteincontainingDnaJ,DnaJ_Cdomains
100	2	267521396	dpca2g172000.840	PREDICTED:gamma-terpinenesynthase,chloroplastic-likeisoformX3(gfam000204)|ProteincontainingTB2_DP1_HVA22,Terpene_synth,Terpene_synth_Cdomains
101	3	3945755	dpca3g176370.840	Basichelix-loop-helixDNA-bindingsuperfamilyproteinisoform1(gfam000946)
102	3	4165387	dpca3g176460.840	Clp,N-terminalprotein5NOC3(gfam000962)|ProteincontainingAAA_2,Clp_N,Exotox-A_targetdomains
103	3	16509970	dpca3g181680.840	Uncharacterizedprotein
104	3	16518726	dpca3g181700.840	PREDICTED:RING-H2fingerproteinATL16-like(gfam000058)|ProteincontainingFANCL_C,Prok-RING_1,Prok-RING_4,zf-ANAPC11,zf-C3HC4,zf-C3HC4_2,zf-C3HC4_3,zf-rbx1,zf-RING-like,zf-RING_11,zf-RING_2,zf-RING_5,zf-RING_UBOX,Zn_ribbon_17domains
105	3	16532095	dpca3g181710.840	PREDICTED:E3ubiquitin-proteinligaseRHA1B-like(gfam005336)|Proteincontainingzf-ANAPC11,zf-C3HC4,zf-C3HC4_2,zf-C3HC4_3,zf-rbx1,zf-RING-like,zf-RING_11,zf-RING_2,zf-RING_5,zf-RING_UBOXdomains
106	3	17635594	dpca3g182280.840	Alpha-1,6-xylosyltransferaseisoform1(gfam000902)|ProteincontainingGlyco_transf_34domains
107	3	29975345	dpca3g186640.840	Rudaputativepolyprotein
108	3	36627361	dpca3g188670.840	sn-glycerol-3-phosphatetransporter(gfam000903)|ProteincontainingMFS_1domains
109	3	51499160	dpca3g192320.840	PREDICTED:proteinECERIFERUM1-likeisoformX2(gfam001080)|ProteincontainingFA_hydroxylase,Wax2_Cdomains
110	3	57367207	dpca3g194260.840	Nodescriptionavailable(gfam002576)|ProteincontainingDUF3615domains
111	3	60817768	dpca3g195760.840	Uncharacterizedprotein(gfam005635)|ProteincontainingPkinase,Pkinase_Tyrdomains
112	3	63998406	dpca3g196730.840	proteincytochromeP45093A2,putative,expressed(gfam000753)|Proteincontainingp450domains
113	3	70834875	dpca3g198920.840	membraneprotein(gfam083074)|ProteincontainingLRR_1,LRR_4,LRR_8domains
114	3	72933205	dpca3g199620.840	Uncharacterizedprotein
115	3	123369281	dpca3g209580.840	FunctionunknownXXXXX(gfam010611)
116	3	125124951	dpca3g209780.840	FunctionunknownXXXXX(gfam010611)|ProteincontainingJIP_LZIIdomains
117	3	131572236	dpca3g210820.840	Uncharacterizedprotein
118	3	138574287	dpca3g211910.840	Uncharacterizedprotein
119	3	138855567	dpca3g211980.840	Descriptionnotavailable
120	3	147076298	dpca3g212930.840	Uncharacterizedprotein
121	3	149476119	dpca3g213210.840	uncharacterizedproteinLOC104596352isoformX1

Sr No	Chr	SNP Position	Gene ID	Gene description
122	3	152794146	dpca3g213710.840	Descriptionnotavailable
123	3	155597939	dpca3g214130.840	FunctionunknownXXXXX(gfam010611)|ProteincontainingJIP_LZII,zf-C4H2domains
124	3	159905183	dpca3g214790.840	Rudaputativepolyprotein
125	3	159973304	dpca3g214820.840	PREDICTED:uncharacterizedproteinLOC100838010isoform1(gfam000334)|ProteincontainingDUF563domains
126	3	178267540	dpca3g217710.840	Nodescriptionavailable(gfam023997)
127	3	183575705	dpca3g218670.840	Retrotransposonprotein,putative,unclassified
128	3	195257204	dpca3g220620.840	PREDICTED:CEN-likeprotein1-like(gfam000416)|ProteincontainingPBPdomains
129	3	197331468	dpca3g220990.840	Descriptionnotavailable
130	3	198193118	dpca3g221270.840	Uncharacterizedprotein(gfam000019)|ProteincontainingAa_transdomains
131	3	212717051	dpca3g224510.840	uncharacterizedN-acetyltransferasep20-likeisoformX1
132	3	214113781	dpca3g224910.840	Nodescriptionavailable(gfam009485)|ProteincontainingDUF1039,PPR,PPR_1,PPR_2,PPR_3,PPR_long,RPM2domains
133	3	237540103	dpca3g230850.840	hypotheticalprotein(gfam017085)|ProteincontainingPkinase,Pkinase_Tyrdomains
134	3	237566752	dpca3g230890.840	50SribosomalproteinL12(gfam000446)|ProteincontainingDUF3181,Ribosomal_60sdomains
135	3	237710932	dpca3g230940.840	PREDICTED:purpleacidphosphatase18-like(gfam000343)|ProteincontainingMetallophos,Metallophos_2,Metallophos_C,Pur_ac_phosph_Ndomains
136	3	238142136	dpca3g231060.840	Uncharacterizedprotein(Fragment)
137	3	238431872	dpca3g231100.840	PREDICTED:CEN-likeprotein1-like(gfam000416)|ProteincontainingPBPdomains
138	3	239100112	dpca3g231170.840	bacterial-likefructose-1,6-bisphosphataseXXXXX(gfam001743)|ProteincontainingSnapin_Pallidin,TPR_MLP1_2domains
139	3	239169662	dpca3g231180.840	ProteincontainingGIT_CCdomains
140	3	239230325	dpca3g231200.840	Predictedprotein(gfam000843)|ProteincontainingAbhydrolase_1,Abhydrolase_6,Hydrolase_4domains
141	3	239342534	dpca3g231230.840	Functionunknown5NOC3(gfam004899)
142	3	239349291	dpca3g231240.840	PREDICTED:ferredoxin-1-like,partial(gfam000741)|ProteincontainingFer2domains
143	3	239361986	dpca3g231250.840	ProteincontainingGlyco_hydro_17domains
144	3	239564544	dpca3g231260.840	hypotheticalprotein(gfam079104)|ProteincontainingCarb_anhydrasedomains
145	3	239767386	dpca3g231350.840	ProteincontainingABC_membrane,ABC_trandomains
146	3	239833532	dpca3g231370.840	3-ketoacyl-CoAsynthase1(gfam000245)|ProteincontainingACP_syn_III,ACP_syn_III_C,Chal_sti_synt_C,FAE1_CUT1_RppAdomains

Sr No	Chr	SNP Position	Gene ID	Gene description
147	3	243699167	dpca3g232280.840	SJCHGC08170protein(Fragment)(gfam002388)|ProteincontainingB_lectin,PAN_2,Pkinase,Pkinase_Tyr,S_locus_glycopdomains
148	3	271904284	dpca3g240510.840	ProteincontainingGlyco_hydro_19domains
149	3	300002014	dpca3g248760.840	PREDICTED:aluminum-activatedmalatetransporter9(gfam000641)|ProteincontainingALMT,FUSC_2domains
150	3	303065527	dpca3g249710.840	ProteincontainingProk-E2_B,UQ_condomains
151	3	316002106	dpca3g255020.840	CTPsynthaseXNOCX(gfam000389)|ProteincontainingJIP_LZII,Myb_DNA-bind_3domains
152	3	322043558	dpca3g258030.840	PREDICTED:inositol-pentakisphosphate2-kinaseisoformX2(gfam002845)|ProteincontainingIns_P5_2-kindomains
153	3	324858012	dpca3g260270.840	dihydroneopterintriphosphatepyrophosphatase(gfam002612)|ProteincontainingNUDIXdomains
154	4	6908911	dpca4g264760.840	Uncharacterized protein (gfam005635) | Protein containing Kinase-like, NAF, Pkinase, Pkinase_Tyr domains
155	4	9423787	dpca4g265930.840	Putative NBS-LRR protein (Fragment) (gfam009993) | Protein containing AAA_22, AAA_33, LRR_4, LRR_8, NACHT, NB-ARC, Rx_N domains
156	4	10194634	dpca4g266220.840	Uncharacterized protein
157	4	10235767	dpca4g266270.840	PREDICTED: cyclin-A2-3-like (gfam000076) | Protein containing Cyclin_C, Cyclin_N domains
158	4	11760424	dpca4g266650.840	Os10g0550300 protein
159	4	12074362	dpca4g266720.840	Spindle assembly abnormal protein 6 (gfam001037) | Protein containing DUF620 domains
160	4	24816491	dpca4g271270.840	Structural maintenance of chromosomes protein (gfam000531) | Protein containing AAA_15, AAA_23, SMC_hinge, SMC_N domains
161	4	24855828	dpca4g271300.840	protein RALFL13 - Rapid ALkalinization Factor RALF family protein precursor, expressed(gfam000422) | Protein containing RALF domains
162	4	24889383	dpca4g271300.840	protein RALFL13 - Rapid ALkalinization Factor RALF family protein precursor, expressed(gfam000422) | Protein containing RALF domains
163	4	24900886	dpca4g271300.840	protein RALFL13 - Rapid ALkalinization Factor RALF family protein precursor, expressed(gfam000422) | Protein containing RALF domains
164	4	25982717	dpca4g271900.840	ABC transporter, ATP-binding protein (gfam051918) | Protein containing ABC_membrane, ABC_tran domains
165	4	31234651	dpca4g273760.840	BTB/POZ domain-containing protein, putative isoform 1 (gfam002904) | Protein containing BTB domains
166	4	39197650	dpca4g276180.840	Monogalactosyldiacylglycerol synthase chloroplastic isoform X1

Sr No	Chr	SNP Position	Gene ID	Gene description
167	4	41164708	dpca4g277060.840	PREDICTED: cytochrome P450 3A30-like (gfam005628) | Protein containing p450 domains
168	4	44260171	dpca4g278000.840	Phosphoinositide phosphatase family protein isoform 4 (gfam001312) | Protein containing Syja_N domains
169	4	44286610	dpca4g278000.840	Phosphoinositide phosphatase family protein isoform 4 (gfam001312) | Protein containing Syja_N domains
170	4	45227929	dpca4g278390.840	Function unknown 5NOC3(gfam006759)
171	4	47125957	dpca4g279040.840	PREDICTED: endoglucanase CX (gfam000218) | Protein containing Glyco_hydro_9 domains
172	4	48575150	dpca4g279470.840	Protein containing Auxin_inducible domains
173	4	48751716	dpca4g279640.840	SAUR56 - auxin-responsive SAUR family member 5NOC3(gfam000056) | Protein containing Auxin_inducible domains
174	4	63781152	dpca4g285180.840	protein CSLD1 - cellulose synthase-like family D, expressed(gfam000149) | Protein containing Cellulose_synt, Glyco_trans_2_3 domains
175	4	67844255	dpca4g286940.840	PREDICTED: S-norcoclaurine synthase 1-like (gfam004806) | Protein containing 2OG-FeII_Oxy, DIOX_N domains
176	4	72939269	dpca4g288860.840	Uncharacterized protein
177	4	77757362	dpca4g290680.840	protein MBTB40 - Bric-a-Brac, Tramtrack, Broad Complex BTB domain with Meprin and TRAF Homology MATH domain(gfam000127) | Protein containing BTB domains
178	4	80514910	dpca4g291550.840	Xyloglucan endotransglucosylase/hydrolase protein 30 (gfam000142) | Protein containing Glyco_hydro_16, XET_C domains
179	4	95901582	dpca4g296030.840	Uncharacterized protein (Fragment)
180	4	100366722	dpca4g297400.840	Protein DOG1-like 3
181	4	100745272	dpca4g297570.840	membrane protein (gfam083074) | Protein containing LRR_1, LRR_4, LRR_6, LRR_8 domains
182	4	139361713	dpca4g306700.840	Tapetum determinant 1, putative isoform 1 (gfam000997)
183	4	140357459	dpca4g306850.840	PREDICTED: RING-H2 finger protein ATL16-like (gfam000058) | Protein containing FANCL_C, Prok-RING_4, zf-C3HC4, zf-C3HC4_2, zf-C3HC4_3, zf-Nse, zf-rbx1, zf-RING_11, zf-RING_2, zf-RING_5, zf-RING_UBOX, Zn_ribbon_17 domains
184	4	156983332	dpca4g309110.840	Uncharacterized protein
185	4	163334205	dpca4g309870.840	Uncharacterized protein (Fragment)
186	4	222400380	dpca4g321770.840	PREDICTED: UPF0481 protein At3g47200-like isoform X2 (gfam000134) | Protein containing DUF247 domains
187	4	229687210	dpca4g323810.840	PREDICTED: LOW QUALITY PROTEIN: autophagy-related protein 13-like (gfam003076) | Protein containing ATG13 domains
188	4	236999746	dpca4g326530.840	Protein containing MS_channel domains
189	4	239821629	dpca4g328400.840	No description available(gfam009157) | Protein containing CCT domains

Sr No	Chr	SNP Position	Gene ID	Gene description
190	5	77862672	dpca5g345960.840	Phosphoglycerate kinase (gfam000993) | Protein containing PGK domains
191	5	85335264	dpca5g348560.840	PREDICTED: LOW QUALITY PROTEIN: DUF246 domain-containing protein At1g04910-like (gfam004650) | Protein containing O-FucT domains
192	5	92008291	dpca5g351430.840	Protein containing bZIP_1, bZIP_2 domains
193	5	93011085	dpca5g351920.840	PREDICTED: endochitinase B-like (gfam000452) | Protein containing Chitin_bind_1, Glyco_hydro_19 domains
194	5	124307775	dpca5g363450.840	ACT domain containing protein, putative, expressed (gfam000638) | Protein containing ACT, ACT_4, ACT_6, LytR_C domains
195	5	130400756	dpca5g365800.840	Cystathionine gamma-synthase (gfam000782) | Protein containing Beta_elim_lyase, Cys_Met_Meta_PP domains
196	5	130405914	**dpca5g365810.840**	**Uncharacterized protein (gfam000272) | Protein containing EMP24_GP25L domains**
197	5	130407209	**dpca5g365810.840**	**Uncharacterized protein (gfam000272) | Protein containing EMP24_GP25L domains**
198	5	143431993	dpca5g371990.840	hypothetical protein (gfam085157) | Protein containing Rhodanese domains
199	5	156277752	dpca5g378870.840	PREDICTED: endo-1,4-beta-xylanase Z-like (gfam000981) | Protein containing CarboxypepD_reg, CBM_4_9, Glyco_hydro_10 domains
200	5	159675841	dpca5g380670.840	No description available(gfam020929) | Protein containing LRRNT_2, LRR_1, LRR_4, LRR_6, LRR_8, Pkinase, Pkinase_Tyr domains
201	5	163662767	dpca5g383470.840	Gb:AAD20392.1 isoform 4 (gfam001476) | Protein containing DNA_pol_A_exo1, DUF639, HRDC, Reticulon domains
202	5	164116688	dpca5g383670.840	Metallophosphoesterase (gfam002485) | Protein containing Metallophos_2 domains
203	5	166754628	dpca5g385370.840	PREDICTED: DIS3-like exonuclease 1 (gfam002076) | Protein containing CSD2, OB_Dis3, PIN_4, RNB, Rrp44_CSD1, Rrp44_S1 domains
204	6	3098168	dpca6g389840.840	PREDICTED: vesicle-associated protein 2-2 isoform X2 (gfam005972) | Protein containing Motile_Sperm domains
205	6	16884709	dpca6g397120.840	PREDICTED: ABSCISIC ACID-INSENSITIVE 5-like protein 2 (gfam000500) | Protein containing bZIP_1, bZIP_2 domains
206	6	20133776	dpca6g398220.840	Proteinase inhibitor I12, Bowman-Birk 5NOC3(gfam004697) | Protein containing Bowman-Birk_leg domains
207	6	40295330	dpca6g404010.840	No description available(gfam020929) | Protein containing Adeno_E3_CR2, B_lectin, DUF3403, PAN_2, Pkinase, Pkinase_Tyr, S_locus_glycop domains
208	6	42671475	dpca6g404920.840	cell division protein FtsY (gfam050174) | Protein containing AAA_17, cobW, MMR_HSR1, SRP54, SRP54_N, SRP_SPB domains

Sr No	Chr	SNP Position	Gene ID	Gene description
209	6	42701420	dpca6g404920.840	cell division protein FtsY (gfam050174) | Protein containing AAA_17, cobW, MMR_HSR1, SRP54, SRP54_N, SRP_SPB domains
210	6	42720239	dpca6g404930.840	single-stranded DNA-binding protein, partial (gfam081648) | Protein containing SSB domains
211	6	42852393	dpca6g404960.840	Tetratricopeptide repeat 4-like protein
212	6	46345601	dpca6g406300.840	PREDICTED: signal recognition particle receptor subunit beta-like (gfam002982) | Protein containing AAA_18, ABC_tran, Arf, ATP_bind_1, FeoB_N, GTP_EFTU, Gtr1_RagA, MMR_HSR1, NACHT, Ras, Roc, SRPRB domains
213	6	59405668	dpca6g409960.840	Aminotransferase, class V family (gfam000739) | Protein containing Aminotran_5 domains
214	6	60135571	dpca6g410360.840	Hexokinase (gfam000571) | Protein containing Hexokinase_1, Hexokinase_2 domains
215	6	81540072	dpca6g416330.840	Uncharacterized protein
216	6	85240212	dpca6g417270.840	Polynucleotidyl transferase, ribonuclease H-like superfamily protein (gfam001006) | Protein containing DNA_pol_A_exo1 domains
217	6	85758862	dpca6g417460.840	Uncharacterized protein
218	6	109188248	dpca6g422300.840	protein OsGH3.9 - Probable indole-3-acetic acid-amido synthetase, expressed(gfam000421) | Protein containing GH3 domains
219	6	124109057	dpca6g425710.840	Protein containing zf-DBF domains
220	6	141365243	dpca6g429700.840	No description available(gfam009048) | Protein containing PPR, PPR_1, PPR_2, PPR_3, PPR_long domains
221	6	141369306	dpca6g429710.840	membrane protein (gfam057120) | Protein containing Gp_dh_N domains
222	6	141556368	dpca6g429730.840	Protein containing zf-CCHC, zf-CCHC_2 domains
223	6	142217225	dpca6g429880.840	Putative transposase related protein
224	6	142296880	dpca6g429900.840	PREDICTED: E3 ubiquitin-protein ligase Os04g0590900-like (gfam011473) | Protein containing Prok-RING_4, zf-ANAPC11, zf-C3HC4, zf-C3HC4_2, zf-C3HC4_3, zf-rbx1, zf-RING_11, zf-RING_2, zf-RING_5, zf-RING_UBOX, Zn_ribbon_17 domains
225	6	145381624	dpca6g430320.840	Protein containing zf-DBF domains
226	6	148448308	dpca6g430790.840	Uncharacterized protein (Fragment)
227	6	148456126	dpca6g430790.840	Uncharacterized protein (Fragment)
228	6	148728038	dpca6g430860.840	Protein containing Cupin_2 domains
229	6	148801439	dpca6g430860.840	Protein containing Cupin_2 domains
230	6	148811602	dpca6g430890.840	Uncharacterized protein (Fragment)
231	6	149700499	dpca6g430960.840	Transcription factor MYB42 5NOC3(gfam000008) | Protein containing Myb_DNA-binding, Myb_DNA-bind_6 domains
232	6	149700872	dpca6g430960.840	Transcription factor MYB42 5NOC3(gfam000008) | Protein containing Myb_DNA-binding, Myb_DNA-bind_6 domains

Sr No	Chr	SNP Position	Gene ID	Gene description
233	6	150525488	dpca6g431090.840	Protein containing ABC_tran_CTD, JIP_LZII, Snapin_Pallidin, Syntaxin_2 domains
234	6	150525524	dpca6g431090.840	Protein containing ABC_tran_CTD, JIP_LZII, Snapin_Pallidin, Syntaxin_2 domains
235	6	151157741	dpca6g431160.840	Uncharacterized protein (Fragment)
236	6	151456453	dpca6g431180.840	Putative ribose-5-phosphate isomerase B (gfam008609) | Protein containing Cupin_2, Cupin_7, LacAB_rpiB domains
237	6	153139197	dpca6g431420.840	Protein containing JIP_LZII domains
238	6	153943117	dpca6g431550.840	Uncharacterized protein
239	6	154131992	dpca6g431590.840	Description not available
240	6	154143999	dpca6g431590.840	Description not available
241	6	154180998	dpca6g431590.840	Description not available
242	6	154482007	dpca6g431630.840	Thioredoxin-like 1-1, chloroplastic
243	6	155130749	dpca6g431720.840	Function unknown XXXXX(gfam010611)
244	6	155143276	dpca6g431720.840	Function unknown XXXXX(gfam010611)
245	6	155521958	dpca6g431750.840	PWWP domain containing protein
246	6	155521965	dpca6g431750.840	PWWP domain containing protein
247	6	155762384	dpca6g431780.840	3-ketoacyl-CoA synthase 1 (gfam000245) | Protein containing ACP_syn_III, ACP_syn_III_C, Chal_sti_synt_C, FAE1_CUT1_RppA domains
248	6	172222184	dpca6g434460.840	Uncharacterized protein
249	6	178041770	dpca6g435350.840	Description not available
250	6	235060792	dpca6g446110.840	No description available(gfam014501) | Protein containing UEV, UQ_con domains
251	6	239715351	dpca6g447430.840	No description available(gfam009485) | Protein containing DUF3368, PPR, PPR_1, PPR_2, PPR_3, PPR_long domains
252	6	244563760	dpca6g448920.840	Anthocyanidin reductase (Fragment)
253	6	248116663	dpca6g450440.840	SJCHGC08170 protein (Fragment) (gfam002388) | Protein containing Pkinase, Pkinase_Tyr domains
254	6	249278794	dpca6g450910.840	Uncharacterized protein
255	6	264330868	dpca6g455740.840	RRNA processing/ribosome biogenesis 5NOC3(gfam006386) | Protein containing RIX1 domains
256	6	273564950	dpca6g459780.840	Uncharacterized protein isoform 1 (gfam003298)
257	6	277493247	dpca6g461620.840	Uncharacterized protein isoform 1 (gfam000613) | Protein containing DUF641 domains
258	6	280066668	dpca6g463530.840	Expressed protein
259	7	84489	dpca7g464710.840	protein TOPBP1B - Similar to DNA replication protein TOPBP1 from
260	7	1497447	dpca7g465640.840	Helicase
261	7	2481826	dpca7g466560.840	PREDICTED: zinc finger protein CONSTANS-LIKE 5-like (gfam000409) | Protein containing CCT
262	7	11929084	dpca7g471370.840	lactate 2-monooxygenase (gfam000833) | Protein containing FMN_dh domains
263	7	11929673	dpca7g471370.840	lactate 2-monooxygenase (gfam000833) | Protein containing FMN_dh domains
264	7	11953597	dpca7g471380.840	lactate 2-monooxygenase (gfam000833) | Protein containing FMN_dh

Sr No	Chr	SNP Position	Gene ID	Gene description
265	7	27440757	dpca7g478020.840	protein scarecrow
266	7	33085807	dpca7g480250.840	Uncharacterized protein (Fragment)
267	7	33954050	dpca7g480480.840	protein endonuclease/Exonuclease/phosphatase family protein
268	7	52388267	dpca7g486590.840	Protein containing DUF630
269	7	67446316	dpca7g491710.840	Protein containing CENP-F_leu_zip
270	7	70828704	dpca7g493000.840	Protein containing Sterile domains
271	7	109320162	dpca7g501110.840	PREDICTED: putative 1-phosphatidylinositol-3-phosphate 5-kinase FAB1D (gfam000980) | Protein containing Cpn60_TCP1
272	7	120107479	dpca7g504050.840	PREDICTED: polyubiquitin-B-like isoform 2 (gfam010872) | Protein containing DUF2407
273	7	121902898	dpca7g504510.840	No description available(gfam020929) | Protein containing LRR_1
274	7	142336899	dpca7g508970.840	Protein containing zf-CCHC
275	7	196098235	dpca7g517900.840	Uncharacterized protein
276	7	199205536	dpca7g518510.840	RNase H domain-containing protein
277	7	207576451	dpca7g520540.840	No description available(gfam020539) | Protein containing AA_kinase
278	7	207578128	dpca7g520540.840	No description available(gfam020539) | Protein containing AA_kinase
279	7	207694797	dpca7g520580.840	4-hydroxybenzoyl-CoA thioesterase (gfam064421) | Protein containing 4HBT_2
280	7	207696790	dpca7g520580.840	4-hydroxybenzoyl-CoA thioesterase (gfam064421) | Protein containing 4HBT_2
281	7	207698929	dpca7g520580.840	4-hydroxybenzoyl-CoA thioesterase (gfam064421) | Protein containing 4HBT_2
282	7	207699230	dpca7g520580.840	4-hydroxybenzoyl-CoA thioesterase (gfam064421) | Protein containing 4HBT_2
283	7	227857258	dpca7g525330.840	PREDICTED: KH domain-containing
284	7	260308833	dpca7g535340.840	Protein containing zf-C2H2_4
285	7	261635550	dpca7g535940.840	Glutaredoxin protein (gfam000154) | Protein containing Glutaredoxin domains
286	7	270998103	dpca7g539850.840	Protein containing Agglutinin
